# PRDM1 Is Associated with Chemoradiotherapy-Associated Enrichment of Adaptive NK Cells in Cervical Cancer

**DOI:** 10.34133/csbj.0092

**Published:** 2026-05-07

**Authors:** Meng Wan, Tangwu Zhong, Wenyang Shi, Jianyu Shen, Wei Zhang, Yizhe Sun

**Affiliations:** ^1^ Department of Radiation Oncology, Beijing Obstetrics and Gynecology Hospital, Capital Medical University, Beijing, China.; ^2^ School of Basic Medicine, Jiamusi University, Jiamusi, Heilongjiang, China.; ^3^ Department of Laboratory Medicine, Division of Pathology, Karolinska Institutet, Stockholm, Sweden.; ^4^ Science for Life Laboratory, Department of Oncology-Pathology, Karolinska Institutet, Stockholm, Sweden.; ^5^ Department of Clinical Science, Karolinska Institutet, Stockholm, Sweden.

## Abstract

Chemoradiotherapy (CRT) induces tumor cell death and remodeling of the tumor immune microenvironment. Adaptive natural killer (aNK) cells, initially characterized in chronic viral infection, are now recognized as important in solid tumors, contributing to antitumor immunity and immune memory. However, the dynamics of aNK cell response to CRT and the regulatory mechanisms behind their activation are not well understood. We analyzed single-cell RNA sequencing data from cervical cancer patients before CRT, after the first CRT fraction, and after the second fraction. We found that CRT markedly enriched aNK cells, with increased cytotoxicity and enhanced virus-defending programs. Among the differentially expressed genes, the transcription factor PRDM1 (PR/SET domain 1, also known as BLIMP-1:B lymphocyte-induced maturation protein 1) was consistently up-regulated in aNK cells after both rounds of CRT. To explore the potential role of PRDM1, we performed in silico perturbation analyses using scTenifoldKnk and CellOracle. These computational simulations predicted reduced effector-associated programs and perturbed metabolic networks following PRDM1 disruption in aNK cells. Moreover, PRDM1 perturbation altered inferred cellular trajectories, opposing the transcriptional shift toward an adaptive NK-associated state, suggesting that PRDM1 may contribute to maintenance of aNK-associated identity and functional features in the CRT-conditioned tumor microenvironment. These findings identify PRDM1 as a candidate regulator associated with aNK cell enrichment, activation-related remodeling, and trajectory-associated changes following CRT, providing insight into immune remodeling during CRT and highlighting PRDM1 as a promising regulatory candidate for future investigation in radiotherapy-induced antitumor immunity.

## Graphical Abstract

**Figure:**
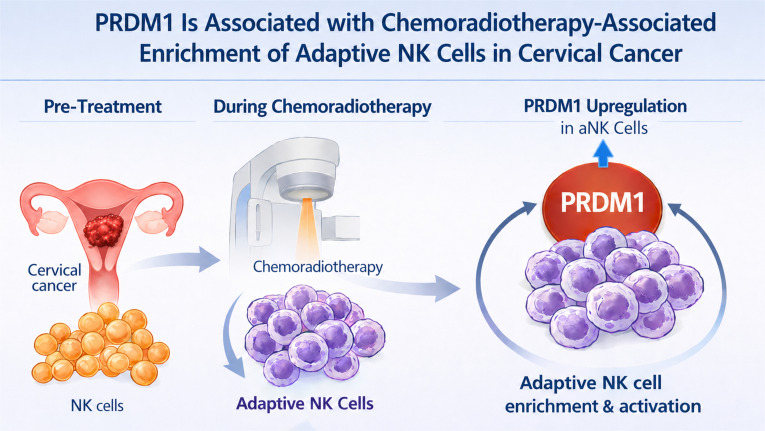

## Introduction

Chemoradiotherapy (CRT) remains a cornerstone treatment for patients with locally advanced cervical cancer and constitutes the standard of care for definitive management of this disease [1,2]. Beyond its direct cytotoxic effects mediated by DNA damage, cell cycle arrest, and apoptosis, accumulating evidence indicates that CRT profoundly remodels the tumor immune microenvironment [3]. Both radiotherapy and chemotherapy can induce immunogenic cell death characterized by the release of damage-associated molecular patterns (DAMPs), including High Mobility Group Box 1 (HMGB1) , adenosine triphosphate (ATP), and surface calreticulin exposure, which promote dendritic cell activation and antigen uptake, thereby enhancing tumor antigen presentation and priming of antitumor T cell responses [4,5]. In parallel, radiation increases the expression of major histocompatibility complex (MHC) class I molecules, death receptors, and stress-induced ligands on tumor cells, augmenting their susceptibility to immune-mediated killing by cytotoxic lymphocytes, including CD8^+^ T cells and natural killer (NK) cells [6,7]. Chemotherapeutic agents further synergize with radiotherapy by modulating immune checkpoint pathways, depleting immunosuppressive cell populations such as regulatory T cells and myeloid-derived suppressor cells, and promoting a pro-inflammatory cytokine milieu within the tumor microenvironment [8–10]. Collectively, these therapy-induced immune alterations drive recruitment, activation, and functional reprogramming of diverse immune cell subsets, ultimately shaping therapeutic efficacy and long-term clinical outcomes [11,12]. Recent computational studies in cervical cancer have likewise highlighted the value of molecular biomarkers for predicting cisplatin-based therapeutic response, underscoring the need to better define treatment-associated biological programs in this disease [13].

NK cells are key innate lymphocytes involved in tumor surveillance and antiviral defense [14]. In the setting of CRT, NK cell states are shaped by therapy-induced inflammatory cues and stress signaling in the tumor microenvironment: radiation can trigger cytosolic DNA sensing and cyclic GMP–AMP synthase–stimulator of interferon genes (cGAS–STING)-dependent type I interferon programs that promote immune activation and can potentiate NK cell activity, while also inducing chemokines that support immune-cell recruitment [15,16]. At the tumor cell level, irradiation can increase expression of immune-relevant surface molecules, including stress-induced ligands such as NKG2D ligands and death receptors, thereby enhancing susceptibility to NK-mediated cytotoxicity, although opposing effects (e.g., altered checkpoint/ligand balance) have also been reported depending on dose and context [17]. Chemotherapy can further modulate NK responses by inducing cellular stress pathways in malignant cells and remodeling suppressive or inflammatory components of the tumor milieu, creating conditions that alter NK activation thresholds and effector outputs [18]. In cervical cancer specifically, single-cell studies of radiochemotherapy/concurrent CRT have demonstrated marked immune-ecosystem remodeling after treatment, underscoring that NK cell activation states and regulatory programs are dynamically rewired during therapy [19]. Commonly, transcriptional regulation is a key determinant of NK cell differentiation, functional maturation, and context-dependent activation within tumors. These therapy-conditioned signals are integrated by core NK transcriptional circuits, with T-bet (TBX21) and Eomes (EOMES) functioning as key maturation checkpoints, and by downstream regulators such as PRDM1 (PR/SET domain 1, also known as BLIMP-1: B lymphocyte-induced maturation protein 1), which controls NK cell maturation and effector cytokine programs [20–22]. In summary, these observations support a model in which CRT reshapes the cervical tumor microenvironment to dynamically reprogram NK cells through inflammatory and stress-associated pathways, altering both tumor susceptibility to NK killing and NK activation thresholds.

Beyond conventional NK cells, a distinct subset termed adaptive or memory-like NK (aNK) cells was first described in the context of chronic viral infection, most prominently human cytomegalovirus (HCMV), where virus-driven selection and expansion generate a highly differentiated NK population with a characteristic receptor and signaling profile [23–25]. These cells are supported by stable epigenetic remodeling and long-term persistence and can display clonal-like expansion with augmented effector potential, particularly in antibody-dependent cellular cytotoxicity and recall-like responsiveness [26–28]. Importantly, the relevance of aNK cells is increasingly recognized beyond infection. Multiple studies and reviews have highlighted their presence in cancer and their potential contribution to antitumor immunity, including enhanced cytotoxic competence and therapeutic promise in solid-tumor settings, especially in contexts where antibody-dependent mechanisms are engaged [29,30]. Nevertheless, whether and how aNK cells dynamically respond to CRT in human cancers has not been systematically explored.

In this study, we analyzed single-cell RNA sequencing (scRNA-seq) data from cervical cancer samples collected before CRT and at sequential time points during treatment to characterize therapy-induced immune remodeling. We observed an enrichment of adaptive NK cells after CRT together with activation of cytotoxic and antiviral transcriptional programs, and identified PRDM1 as a prominently induced transcriptional regulator in this population. To probe the functional relevance of PRDM1, we applied complementary in silico perturbation approaches to infer regulatory and trajectory-level consequences of PRDM1 disruption. Together, these analyses suggest that PRDM1 may contribute to CRT-associated adaptive NK cell responses and provide a framework for understanding transcriptional control of therapy-driven NK cell adaptation in cervical cancer.

## Results

### CRT is associated with enrichment of aNK cells and induction of PRDM1 in aNK cells

**Fig. 1.**
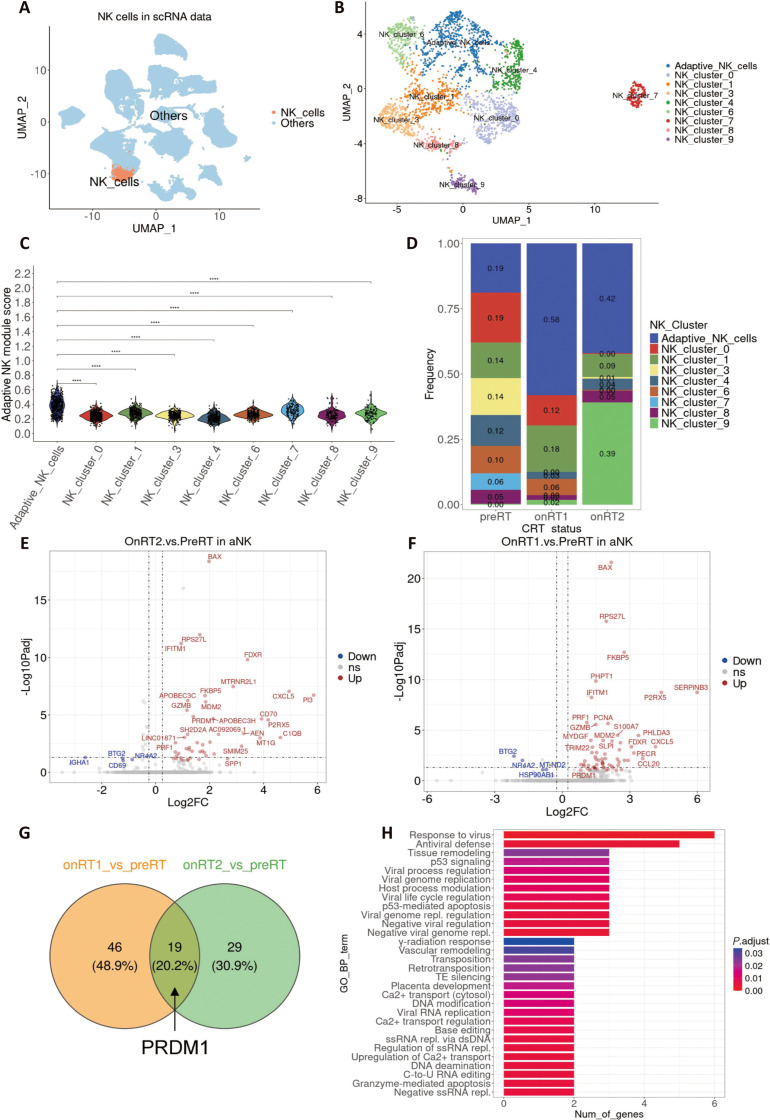
Chemoradiotherapy (CRT) induces up-regulation of the transcription factor PRDM1 in aNK cells. (A) UMAP visualization shows natural killer (NK) cells separated from the total cells. (B) UMAP visualization of NK cell subclusters derived from the extracted NK cell population. (C) Violin plot displaying the distribution of aNK cell module scores across all clusters, with pairwise Mann–Whitney–Wilcoxon tests performed using aNK cluster as the reference group. Statistical significance is denoted by: ****P < 0.0001. (D) Stacked bar plots show the relative frequencies of NK subsets in pretreatment (preRT), first-time CRT (onRT1), and second-time CRT (onRT2). (E and F) Volcano plots depict differentially expressed genes in aNK cells comparing onRT2 vs. preRT (E) and onRT1 vs. preRT (F). (G) A Venn diagram highlights shared up-regulated genes between the 2 comparisons, including PRDM1. (H) Gene Ontology (GO) analysis of overlapping genes reveals enriched biological processes.

We analyzed publicly available scRNA-seq data from cervical cancer patients in the Gene Expression Omnibus (GEO) dataset GSE297041 [31], including samples collected before CRT (preRT), after the first CRT fraction (onRT1), and after the second fraction (onRT2). From the global immune landscape, NK cells were first identified and extracted based on NK features and visualized on a Uniform Manifold Approximation and Projection (UMAP) embedding, showing a distinct NK compartment separated from other immune populations (Fig. 1A). The NK subset analyzed in this study comprised 2,842 NK cells from 10 patients, including 2,423, 112, and 307 cells from preRT, onRT1, and onRT2, respectively. Within this subset, 650 aNK cells were identified, including 456, 65, and 129 cells across the 3 treatment time points, indicating an uneven but informative longitudinal distribution of NK and adaptive NK populations. Next, to annotate all NK cell clusters (Fig. 1B), we applied scType [32] to identify the cluster most consistent with aNK features, using marker genes compiled from 2 sources: bulk RNA-seq of late-mature aNK versus early-mature cNK cells from human peripheral blood [33,34] and transcriptomic profiles of ovarian tumor-infiltrating NK cells [29] (Table S1). To quantify the aNK phenotype across NK subclusters, we calculated an aNK module score and compared its distribution among clusters. The aNK population displayed higher module scores than other NK subsets, supporting its identity as a transcriptionally distinct NK state within the dataset (Fig. 1C). We then examined how NK subset composition changed across CRT time points. Notably, the relative frequency of aNK cells increased following CRT, with enrichment already apparent after the first fraction and persisting after the second fraction (Fig. 1D), indicating a rapid and sustained therapy-associated shift in NK cell composition.

To characterize transcriptional remodeling of aNK cells during CRT, we performed differential expression analyses comparing onRT2 vs. preRT and onRT1 vs. preRT within the aNK compartment. Both comparisons revealed broad CRT-associated transcriptional changes, including induction of cytotoxic and interferon/virus-response-related genes (Fig. 1E and F). A set of up-regulated genes was shared between onRT1 and onRT2 relative to preRT, suggesting a conserved CRT-induced aNK program across treatment time points (Fig. 1G). Among these overlapping genes, PRDM1 was prioritized for follow-up analysis not solely because it was differentially expressed, but because it represented the strongest convergence of recurrent induction across treatment time points, known immunological relevance, and consistency with our prior observations in tumor-infiltrating adaptive NK cells. In particular, PRDM1 has been implicated in lymphocyte differentiation, memory- and exhaustion-related programs, and NK cell maturation and functional regulation. Importantly, PRDM1 emerged as a consistently up-regulated transcription factor in both comparisons with high expression in the aNK cell cluster (Fig. S1A). Gene Ontology (GO) enrichment analysis of overlapping up-regulated genes highlighted immune activation and antiviral-response pathways consistent with enhanced effector programs in CRT-associated aNK cells (Fig. 1H). Together, these results indicate that CRT is accompanied by enrichment of aNK cells and coordinated activation of cytotoxic/antiviral transcriptional programs, with PRDM1 as a recurrently induced regulator in aNK cells.

### Pseudotime analysis reveals dynamic state transitions and PRDM1 induction along the CRT-associated NK trajectory

**Fig. 2.**
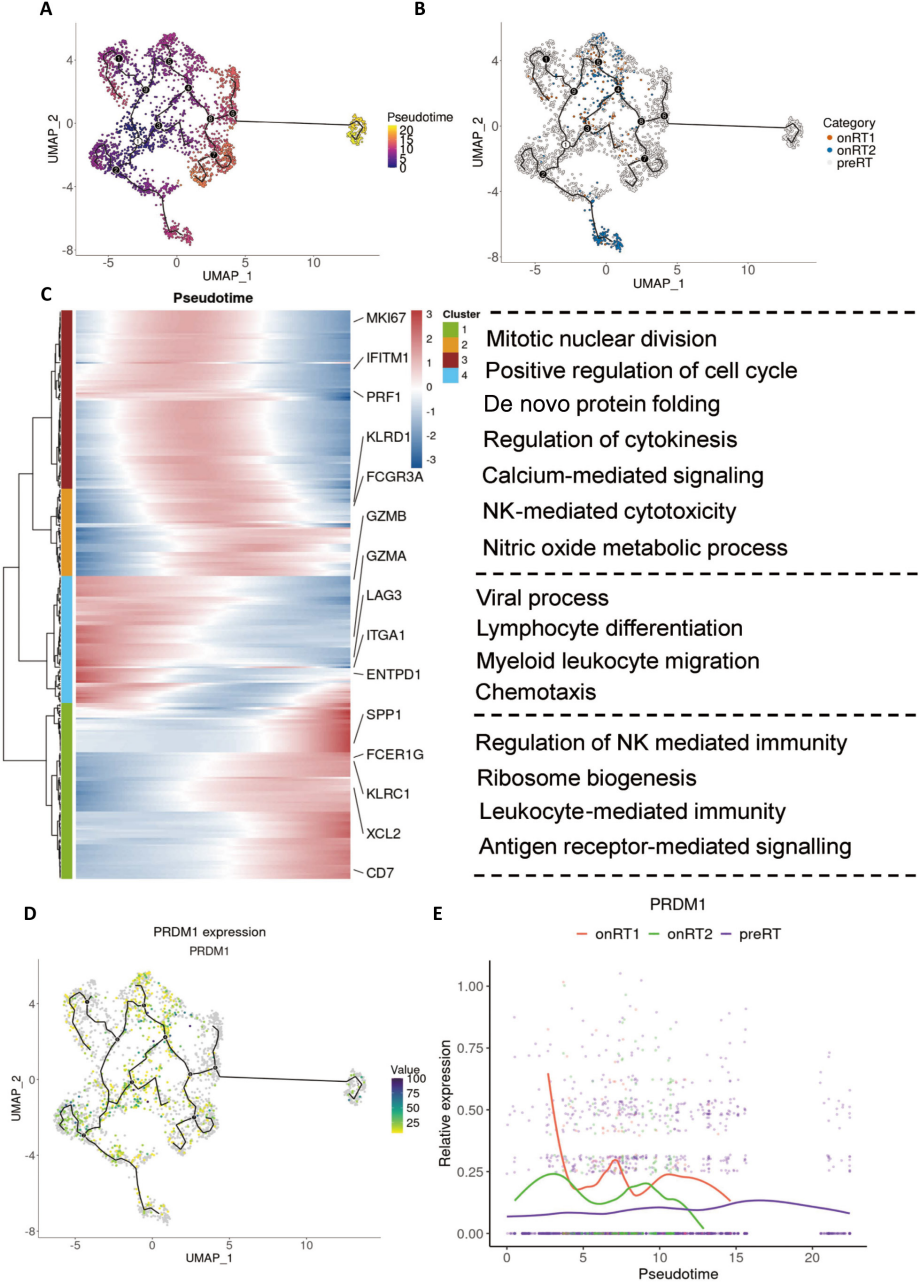
Pseudotime trajectory analysis reveals dynamic transcriptional changes of NK cells. (A) UMAP embedding overlaid with pseudotime values illustrates the inferred developmental trajectory of NK cells, with color indicating progression along pseudotime. (B) Cells are colored by treatment condition (preRT, onRT1, and onRT2) and projected onto the trajectory to show their distribution along the developmental path. (C) Heatmap displays the modules of dynamically expressed genes along pseudotime, with representative genes labeled and major enriched GO biological processes summarized on the right. (D) UMAP visualization of PRDM1 expression demonstrates its spatial distribution along the trajectory, with color intensity indicating expression levels. (E) Scatter plot shows PRDM1 expression dynamics across pseudotime in preRT, onRT1, and onRT2 conditions, with fitted curves indicating overall trends.

To further characterize the CRT-associated transcriptional dynamics within NK cells, we performed pseudotime trajectory analysis on the NK cell compartment. Mapping cells onto the UMAP embedding with inferred pseudotime values revealed an ordered continuum spanning multiple NK states, suggesting an inferred transcriptional continuum in which the aNK cell cluster occupied one terminal region of the trajectory (Fig. 2A). Along the trajectory, cells from onRT1 and onRT2 were preferentially distributed toward terminal pseudotime states (Fig. 2B).

Consistent with this progression, genes that varied dynamically along pseudotime organized into modules with coordinated expression patterns, capturing distinct phases of NK cell reprogramming (Fig. 2C). Early-to-intermediate pseudotime segments were characterized by enrichment of processes related to cell cycle and protein folding, whereas later segments were associated with immune functional programs, including cytokine regulation, NK-mediated cytotoxicity, and antiviral response pathways (Fig. 2C). This pattern suggests progressive transcriptional remodeling of NK cells along pseudotime, accompanied by coordinated changes in functional gene modules.

Given that PRDM1 was consistently induced in aNK cells after CRT in differential expression analyses, we next examined PRDM1 expression along the inferred NK trajectory. PRDM1 expression was spatially enriched along specific regions of the trajectory on UMAP (Fig. 2D) and displayed stage-dependent expressions across pseudotime (Fig. 2E). PRDM1 expression exhibited a pseudotime-dependent pattern and differed by treatment time points. Compared with preRT, on-treatment cells (onRT1 and onRT2) showed higher PRDM1 expression over early-to-intermediate pseudotime, with a pronounced increase in onRT1. In contrast, preRT cells maintained relatively low and stable PRDM1 levels across the trajectory, indicating that PRDM1 up-regulation is associated with the treatment-enriched NK states along pseudotime (Fig. 2E). Together, these analyses suggest that CRT is associated with a treatment-related transcriptional shift in NK cell states along pseudotime and that PRDM1 induction occurs within this inferred trajectory and that PRDM1 induction occurs within this trajectory.

### GRN and regulon analysis highlight PRDM1 as a prominent inferred regulatory candidate associated with aNK cells and CRT

**Fig. 3.**
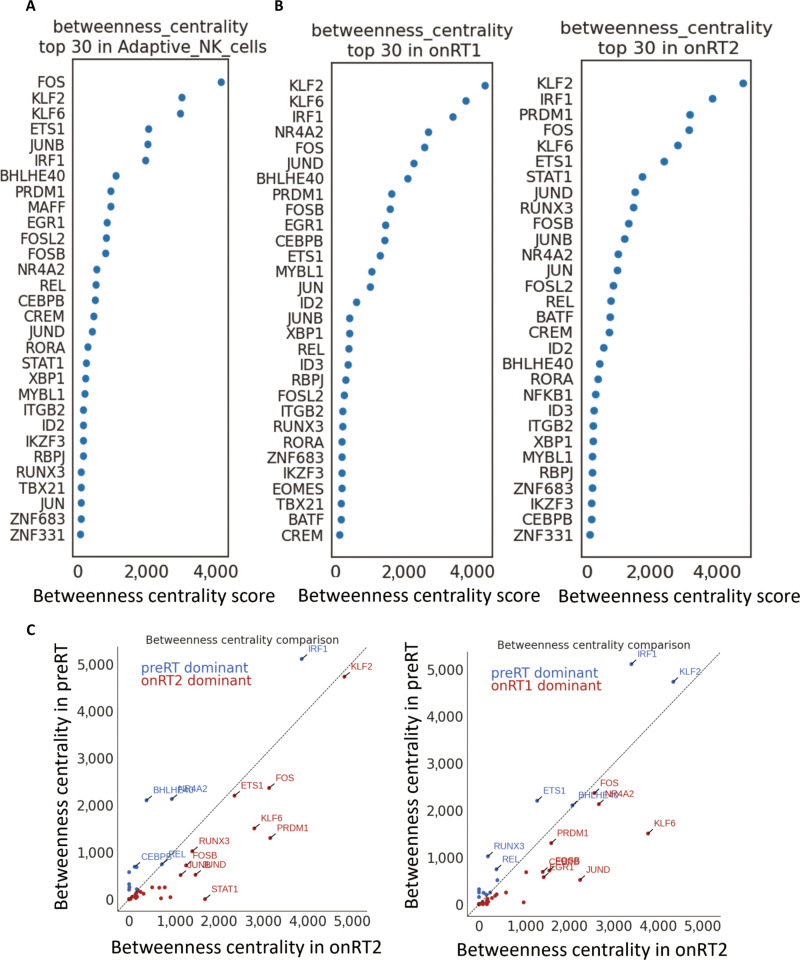
Gene regulatory network (GRN) analysis highlights prominent inferred regulatory candidates in aNK cells during CRT. (A and B) Dot plots show the top 30 transcription factors ranked by betweenness centrality in GRNs inferred from adaptive NK cells (A), onRT1 cells (B, left), and onRT2 cells (B, right). Higher betweenness centrality indicates that a transcription factor occupies a more topologically connected bridging position within the inferred regulatory network. (C) Scatter plots compare betweenness centrality values between preRT and onRT2 (left) and between preRT and onRT1 (right). Each dot represents one transcription factor. Points above the diagonal indicate higher centrality in preRT, whereas points below the diagonal indicate higher centrality in the corresponding on-treatment network. Highlighted labels indicate selected transcription factors with comparatively large shifts in network centrality across conditions.

To identify key transcriptional regulators of aNK cell programs and to assess how these regulatory networks change during CRT, we performed gene regulatory network (GRN) analysis using CellOracle and ranked transcription factors by betweenness centrality, a topological metric reflecting how strongly a node bridges multiple regulatory modules within the inferred network. Higher betweenness centrality indicates that a transcription factor occupies a more connected bridging position in the inferred GRN. In the GRN constructed from aNK cells, centrality ranking highlighted a core set of regulatory hubs dominated by immediate-early and immune-associated transcription factors, including FOS, KLF2, KLF6, IRF1, and others. PRDM1 was also among the top-ranked transcription factors, suggesting that it represents a structurally prominent inferred node within the aNK regulatory network (Fig. 3A).

We next assessed whether the regulatory architecture was altered across CRT stages by constructing condition-specific GRNs for on-treatment samples. Betweenness centrality ranking of the onRT1 and onRT2 GRNs showed a reordered set of hub regulators compared with baseline (Fig. 3B). Notably, PRDM1 remained among the highly ranked transcription factors in both onRT1 and onRT2, indicating that it represents a prominent inferred node in the treatment-associated network architecture in addition to being transcriptionally induced (Fig. 3B). PRDM1 was likewise prioritized by other centrality metrics, including degree and eigenvector centrality (Fig. S2A and B).

To further quantify treatment-related changes in network structure, we directly compared transcription factor betweenness centrality values between preRT and on-treatment states. These comparisons identified PRDM1 as one of the regulators with increased centrality after treatment (onRT-dominant) and those with higher centrality at baseline (preRT-dominant) (Fig. 3C). PRDM1 was among the transcription factors showing treatment-associated prominence in these comparisons, supporting its repeated prioritization across multiple inferred network analyses. Together, these results indicate that CRT is accompanied by measurable rewiring of the aNK GRN and highlight PRDM1 as a recurrent, treatment-associated inferred regulatory candidate across onRT1 and onRT2 states.

To provide orthogonal support for this inference, we also performed regulon analysis using pySCENIC across NK clusters. Adaptive NK cells showed coordinated enrichment of multiple TF regulons, among which PRDM1(+) was prominently activated together with STAT2(+), JUNB(+), EGR1(+), and FOS(+) (Fig. S1B). Compared with other NK clusters, adaptive NK cells displayed the strongest overall regulon activity pattern, supporting that PRDM1 is part of a broader adaptive NK-associated transcriptional regulatory program rather than an isolated signal derived from a single network-inference framework.

### CellOracle in silico perturbation predicts PRDM1-dependent trajectory dynamics in NK cells and aNK cells

**Fig. 4.**
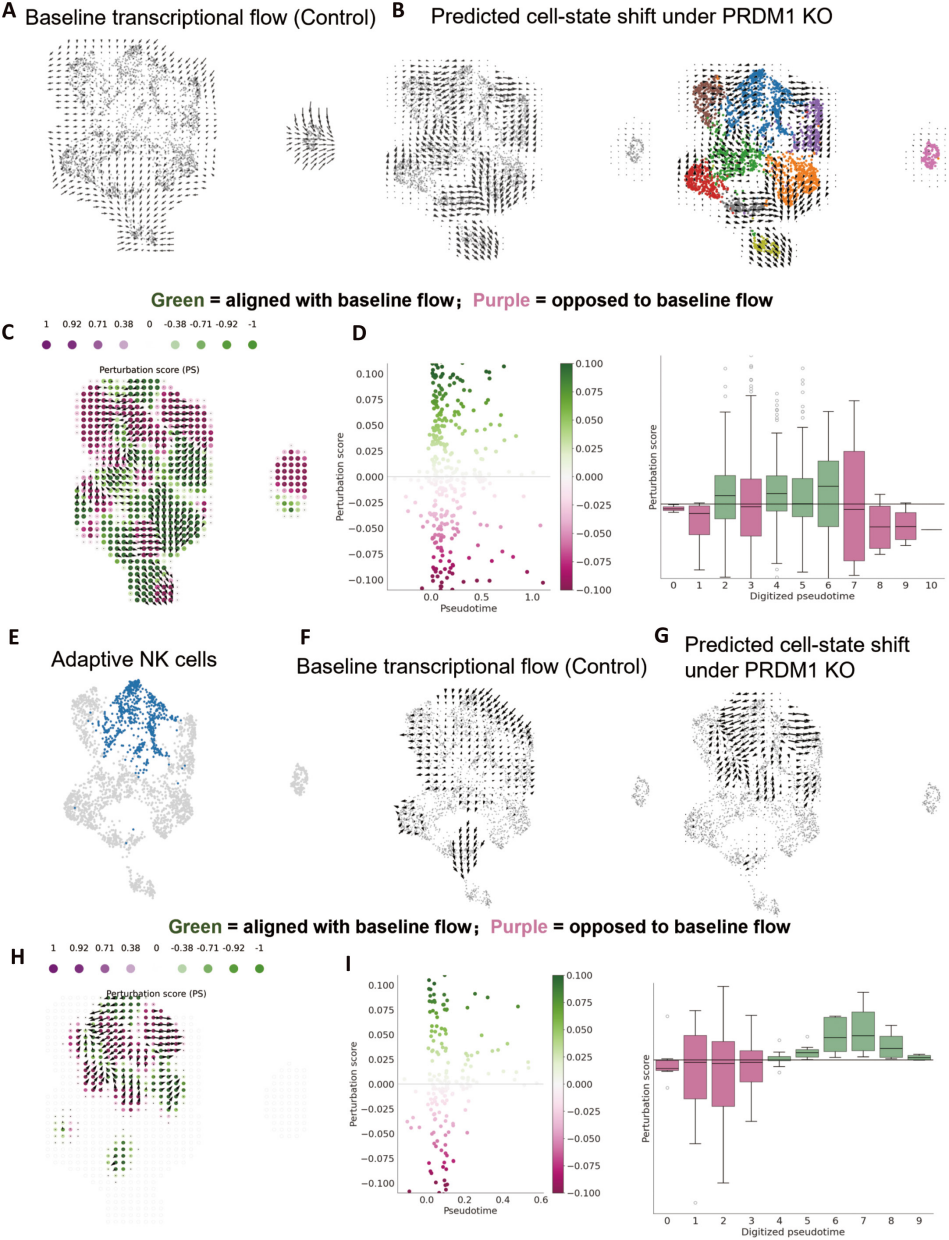
CellOracle-based in silico knockout (KO) of PRDM1 predicts altered inferred transcriptional flow and state-transition dynamics in aNK cells. (A) Baseline transcriptional flow inferred under control conditions, shown as vector fields over the NK cell UMAP. Arrow direction indicates the inferred direction of transcriptional state movement, and arrow length reflects the magnitude of the inferred flow. (B) Predicted cell-state shifts following PRDM1 in silico knockout, projected onto the NK cell UMAP. (C) Perturbation score (PS) projected onto the NK cell UMAP. PS was defined as the inner product between the perturbation vector and the baseline transcriptional flow vector at each grid location. Positive values (green) indicate alignment with the baseline flow, whereas negative values (purple) indicate opposition to the baseline flow. (D) PS along pseudotime shown as a scatter plot across continuous pseudotime (left) and as box plots across discretized pseudotime bins (right). (E) UMAP visualization highlighting adaptive NK cells within the NK cell population. (F and G) Baseline transcriptional flow under control conditions (F) and predicted cell-state shifts following PRDM1 in silico knockout (G), shown specifically for the adaptive NK cell cluster. (H) PS projected onto the adaptive NK cell UMAP. (I) PS dynamics along pseudotime within the adaptive NK cell cluster, shown as a scatter plot across continuous pseudotime (left) and as box plots across discretized pseudotime bins (right).

Given the induction and network centrality of PRDM1 in CRT-associated aNK cells, we next used CellOracle [35] to perform in silico perturbation and evaluate how PRDM1 disruption reshapes inferred cell-state dynamics. Under control conditions, baseline transcriptional flow vectors overlaid on the NK UMAP revealed coherent directional patterns across NK cell states, consistent with an inferred transcriptional ordering in the NK compartment (Fig. 4A). Simulating PRDM1 knockout (KO) produced a marked change in the predicted direction and magnitude of cell identity shifts across the embedding (Fig. 4B), suggesting that PRDM1 may influence the inferred dynamics of NK state transitions.

To quantify perturbation effects relative to the baseline developmental flow, we computed a perturbation score (PS) and projected it onto the NK UMAP. PS was defined as the inner product (dot product) between the 2-dimensional (2D) perturbation-simulation vector and the baseline developmental-flow vector at each location, capturing both vector direction and magnitude; thus, positive PS indicates alignment with the baseline transcriptional flow, whereas negative PS indicates opposition to that flow (Fig. 4C). When PS was examined along pseudotime, PRDM1 KO showed pseudotime-dependent effects on total NK dynamics, with the strongest contradictory impact at late pseudotime where PRDM1 perturbation was associated with the opposite direction to the natural developmental flow (Fig. 4D).

We then focused on the aNK compartment highlighted on the UMAP (Fig. 4E). Within the aNK cluster, baseline developmental flow vectors remained directional (Fig. 4F), whereas PRDM1 KO simulation altered the predicted identity shift patterns (Fig. 4G). Consistently, PS projected onto the aNK UMAP demonstrated localized regions of positive and negative perturbation effects (Fig. 4H), and pseudotime analysis restricted to aNK cells showed a clear shift in PRDM1 KO effects across the continuum, with negative PS values at early pseudotime indicating opposition to the developmental flow (predicted blockade of differentiation) and positive PS values at later pseudotime indicating alignment with the flow (predicted promotion of differentiation) (Fig. 4I). Together, these CellOracle simulations suggest a model in which PRDM1 may contribute to the inferred transcriptional flow and state-transition dynamics of NK cells, with pronounced and stage-dependent effects within the aNK compartment.

### scTenifoldKnk virtual KO of PRDM1 predicts broad downstream network perturbation with prominent metabolic signatures in aNK cells

**Fig. 5.**
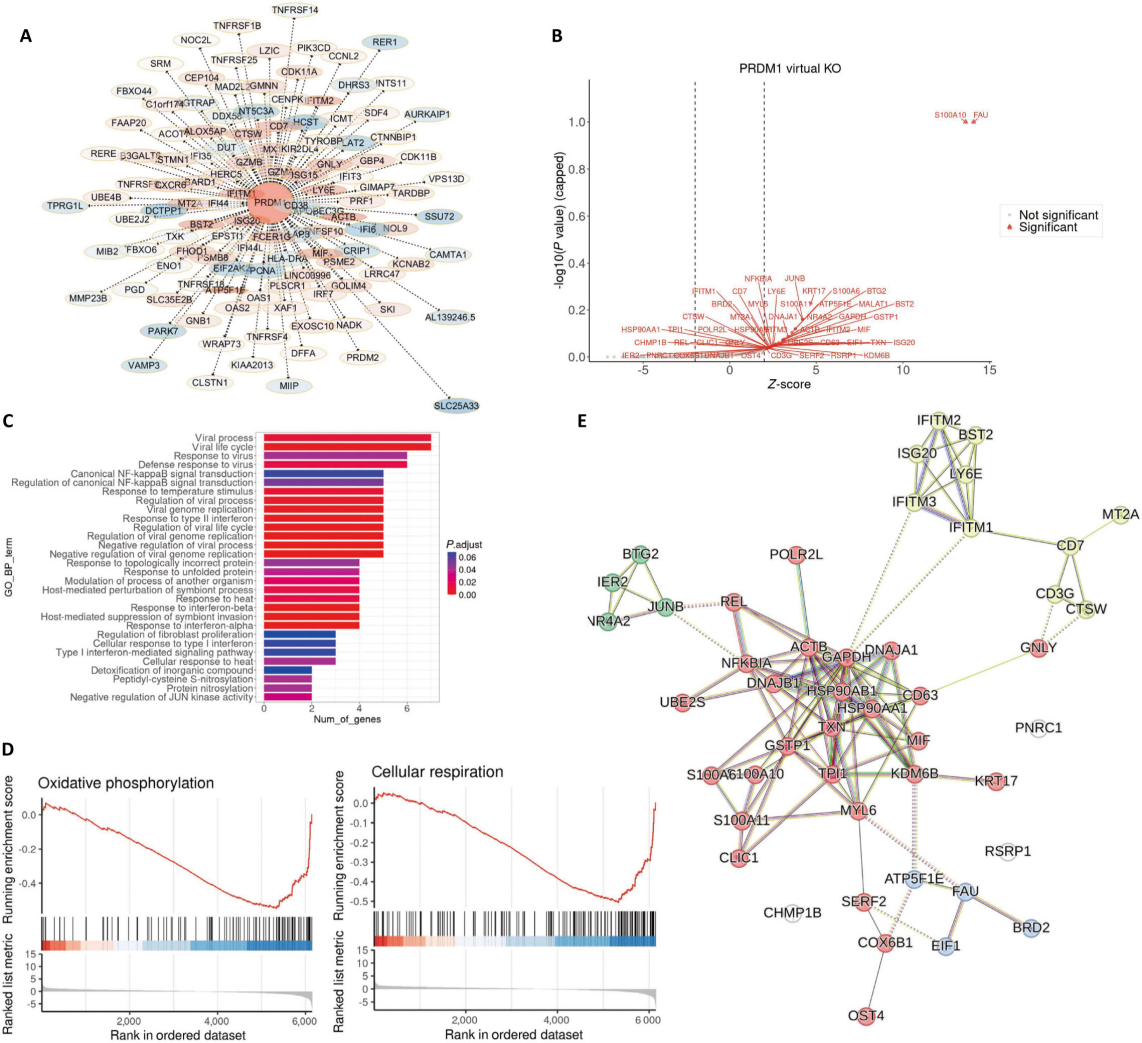
scTenifoldKnk-based in silico KO of PRDM1 reveals downstream regulatory and functional effects in aNK cells. (A) Gene regulatory network (GRN) visualization generated in Cytoscape highlights PRDM1-centered perturbed genes, with nodes representing affected genes and colored according to their *Z*-scores. (B) Volcano plot shows genes most affected by PRDM1 virtual KO, with significantly perturbed genes highlighted. (C) Bar plot showing GO enrichment results of the top genes affected by PRDM1 virtual KO. (D) Gene set enrichment analysis (GSEA) demonstrates significant enrichment of oxidative phosphorylation and cellular respiration pathways upon PRDM1 perturbation. (E) Protein–protein interaction (PPI) network of the top genes affected by PRDM1 virtual knockout, constructed using STRING and visualized to show functional connectivity among perturbed genes.

To further define downstream programs regulated by PRDM1 in aNK cells, we performed in silico PRDM1 KO using scTenifoldKnk [36] and quantified gene-level perturbation effects. The resulting perturbed gene set was mapped onto a PRDM1-centered regulatory context, visualized in Cytoscape [37], highlighting PRDM1-associated connectivity among affected targets and their relative perturbation strength (Fig. 5A). Gene-level perturbation statistics showed a subset of genes exhibiting strong deviation upon PRDM1 virtual KO, as summarized by *Z*-scores and significance in the volcano plot (Fig. 5B), suggesting widespread predicted transcriptional consequences of PRDM1 disruption.

We next asked which biological processes were overrepresented among the top perturbed genes. GO enrichment analysis revealed that the scTenifoldKnk-derived gene set was strongly enriched for immune response-related programs, particularly antiviral/defense-associated processes (Fig. 5C). In parallel, gene set enrichment analysis (GSEA) of the scTenifoldKnk network *Z*-score ranked gene list highlighted a dominant mitochondrial bioenergetic signature, with enriched GO terms related to electron transport chain activity, respiratory complex assembly, and ATP synthesis coupled transport, and concordant Kyoto Encyclopedia of Genes and Genomes (KEGG) enrichment for oxidative phosphorylation (Fig. S3A and B). Importantly, oxidative phosphorylation and cellular respiration showed negative enrichment, suggesting that PRDM1 virtual KO is associated with a predicted shift of these pathways toward the negative end of the ranked perturbation profile, consistent with perturbation of mitochondrial metabolic programs following PRDM1 disruption (Fig. 5D). Finally, following scTenifoldKnk-based PRDM1 virtual KO, we constructed a protein–protein interaction (PPI) network using STRING and performed network clustering, which resolved 4 densely connected modules among the top perturbed genes (Fig. 5E). The first module comprised canonical interferon-stimulated/antiviral factors, including IFITM1/2/3, ISG20, BST2, and LY6E, forming a tightly interconnected cluster consistent with coordinated regulation of antiviral programs. A second module captured cytotoxic and lymphocyte effector features, with CD7, CD3G, CTSW, and GNLY connected to additional stress-associated components (e.g., MT2A), indicating perturbation of effector-associated circuitry. A third module was enriched for immediate-early and inflammatory transcriptional regulators, including REL, NFKBIA, JUNB, NR4A2, BTG2, IER2, and UBE2S, consistent with rewiring of NF-κB/AP-1-linked response networks upon PRDM1 perturbation. The fourth module was dominated by metabolic and proteostasis-related genes, centered on chaperones and redox/stress proteins such as HSP90AA1/HSP90AB1, DNAJA1/DNAJB1, TXN, GSTP1, GAPDH, ACTB, TPI1, and MIF, and extending to mitochondrial/translation-associated components including ATP5F1E, COX6B1, EIF1, and FAU, supporting coordinated disruption of bioenergetic and protein homeostasis pathways. Notably, several connector nodes bridged these modules, indicating that PRDM1 perturbation is predicted to jointly impact antiviral signaling, inflammatory regulation, cytotoxic effector programs, and metabolic/proteostasis networks within aNK cells.

## Methods

### Data source, preprocessing, and NK cell subclustering

Single-cell RNA-seq data from cervical cancer patients were obtained from GEO (GSE297041) and analyzed using a preprocessed Seurat object saved as an R data serialization file (RDS) file. The analyzed cohort included 18 primary tumor samples from 10 cervical cancer patients treated with definitive chemoradiation, with longitudinal biopsies collected before treatment (preRT), 1 to 2 weeks after treatment initiation (onRT1), and 2 to 3 weeks after treatment initiation (onRT2). Because this study was based on a publicly available dataset, detailed clinicopathologic and treatment information was limited in the accompanying metadata. NK cells were extracted from the global dataset based on the cell identity label assigned by the cell annotation. A total of 2,842 NK cells from 10 patients were retained for downstream analyses, including 2,423, 112, and 307 cells from preRT, onRT1, and onRT2, respectively. Within the NK compartment, 650 adaptive NK cells were identified, including 456, 65, and 129 cells from preRT, onRT1, and onRT2, respectively. The distribution of cells across treatment time points and individual patients was uneven, with not all patients contributing cells at all longitudinal time points. NK cells were extracted from the global dataset based on the cell identity label assigned by the cell annotation. For preprocessing within the NK compartment, mitochondrial genes were defined by the MT- prefix and the mitochondrial transcript fraction was calculated per cell; expression values were then normalized, 3,000 highly variable genes were selected, and the data were scaled with regression of mitochondrial content. Dimensionality reduction was performed by PCA, and the NK subset was subclustered by constructing a nearest-neighbor graph using the first 17 principal components followed by graph-based clustering at resolution 0.5. The resulting NK subclusters were visualized using a UMAP embedding generated from the same principal components. All the R processing was performed using Seurat (v5.3.0) [38].

### Cell-type annotation of NK cells and identification of aNK cells

Cell-type identities in the full cervical cancer scRNA-seq dataset were assigned using SingleR (v2.10.0) [39]. Briefly, the raw count matrix was queried against curated human cell atlas references, and each cell was labeled by the best-matching immune or primary cell-type profile; these predicted labels were then visualized on the global UMAP and summarized across unsupervised clusters. To derive a robust cluster-level annotation, we computed the contingency table between predicted labels and clustering assignments and assigned each cluster the most frequent predicted label, which was then propagated back to all cells as a cluster annotation.

For finer annotation within the NK compartment and specific identification of aNK cells, we applied a marker-based scoring approach using scType [32]. Positive and negative marker gene sets were scored on the scaled expression matrix of the NK subset, and scores were summarized at the cluster level to determine the best-supported NK subtype for each cluster. The subtype with the highest aggregated score was assigned as the annotation, while clusters with low-confidence support, defined as a top score below 25% of the cluster cell number, were labeled as Unknown.

### Differential expression and enrichment analyses

aNK cells were extracted from the NK subset based on the assigned NK subtype annotation and grouped by treatment time point (preRT, onRT1, and onRT2). Differential expression was performed within the aNK compartment by comparing onRT1 versus preRT and onRT2 versus preRT using a Wilcoxon rank-sum test, including genes detected in at least 10% of cells in either group. Given the uneven numbers of aNK cells across patient–time point combinations and the incomplete longitudinal sampling across patients, this cell-level Wilcoxon rank-sum framework was used as an exploratory screening approach and did not explicitly model within-patient correlation across repeated samples. Because gene-level differential expression in sparse scRNA-seq data was used here as an exploratory screening step to retain potentially informative transcriptional signals, genes were categorized as up-regulated or down-regulated for visualization and downstream summaries using an adjusted *P* value threshold of 0.1 together with an absolute log2 fold-change cutoff of 0.5. Volcano plots were generated with selected significant genes labeled, including PRDM1.

To identify conserved treatment-associated induction signatures, up-regulated gene sets from onRT1 versus preRT and onRT2 versus preRT were intersected and visualized using set-overlap plots. Functional enrichment analysis was then performed on the overlapping up-regulated genes using GO biological process (GO-BP) over-representation testing with Benjamini–Hochberg correction. Because pathway-level enrichment represents a higher-level biological interpretation step and can be sensitive to the composition of the input gene list, stricter significance criteria were applied at this stage to reduce over-interpretation of higher-order biological themes. Accordingly, enriched terms were defined using thresholds of adjusted *P* < 0.01 and *q* < 0.05, ranked by adjusted *P* value, and visualized using bar plots of the top pathways.

### Regulon analysis using pySCENIC

To provide orthogonal support for transcription factor activity inference, regulon analysis was performed using pySCENIC [40,41] on the cervical NK cell subset. Briefly, the Seurat-derived NK cell count matrix and metadata were converted into AnnData format and exported as a loom file for pySCENIC analysis. GRN inference was first performed using GRNBoost2 [42] with the human transcription factor reference list to generate candidate TF–target adjacencies. Next, motif enrichment pruning was carried out using the hg38 cisTarget ranking database and motif annotation table to identify high-confidence regulons, with dropout masking enabled. Regulon activity in individual cells was then quantified using AUCell, and the resulting regulon activity matrix was imported for downstream analysis. To compare cluster-level regulon activity patterns across NK subtypes, regulon specificity scores (RSSs) were calculated based on NK cell subtype annotations, and *Z*-score-transformed RSS values were visualized as clustered heatmaps. Selected TF regulons, including PRDM1(+), were further displayed to assess their relative enrichment in aNK cells.

### Pseudotime trajectory analysis

Trajectory inference was performed on the NK cell subset using monocle3 (v1.4.26) [43] single-cell pseudotime framework. The count matrix and corresponding cell metadata from the NK subset were converted into a trajectory object, followed by dimensionality reduction and construction of a UMAP embedding. To ensure consistency with the clustering-based visualization used throughout the study, the trajectory UMAP coordinates were replaced with the UMAP embedding previously computed in the Seurat NK analysis. A principal graph was then learned on the UMAP space (without partitioning), and cells were ordered along the graph to assign pseudotime values. To orient the trajectory in a biologically informed manner, the root was assigned to the region/principal node most enriched for preRT cells, because preRT represented the earliest observed treatment time point and therefore provided the most appropriate baseline reference for pseudotemporal ordering in this longitudinal treatment-associated context. Pseudotime and treatment time points were visualized on the trajectory, and PRDM1 expression was projected onto the same embedding. To identify genes with significant spatial dependence along the trajectory, graph-based tests were performed and significant genes (*q* value < 0.05) were grouped into coexpression modules; module-level expression patterns were summarized across NK cell types and visualized as heatmaps. For higher-level interpretation, pseudotime-ordered expression matrices of significant genes were smoothed, *Z*-scored, binned along pseudotime, and clustered into 4 major gene modules, followed by GO-BP enrichment analysis for each module using multiple-testing correction.

### GRN inference and centrality analysis

GRNs were inferred from the NK cell AnnData object using CellOracle with a human promoter-based prior GRN as the base reference. Raw (unscaled) count matrices were used as input, and cells were grouped either by NK subtypes to generate subtype-specific GRNs or by treatment time points (category; preRT, onRT1, onRT2) to generate condition-specific GRNs. After dimensionality reduction and k-nearest-neighbor (kNN)-based imputation, GRN edges were estimated within each group and filtered using a stringent significance cutoff (*P* < 0.001), retaining the top-ranked edges by absolute coefficient (2,000 links). This fixed edge-retention threshold was applied to reduce low-confidence links arising from sparse single-cell measurements while preserving a comparable dominant inferred regulatory structure across NK subtypes and treatment time points for downstream topological comparison. For each inferred GRN, network topology metrics were computed and transcription factors were ranked by centrality-based network scores, including betweenness centrality, degree centrality, and eigenvector centrality, to prioritize hub regulators. Centrality rankings were visualized as score-rank plots within aNK and within each CRT stage, and treatment-associated network rewiring was assessed by pairwise comparison of transcription factor betweenness centrality between on-treatment (onRT1 or onRT2) and baseline (preRT) networks, highlighting regulators with stage-enriched connectivity.

### CellOracle in silico transcription factor perturbation

To assess the predicted functional impact of PRDM1 on NK-state dynamics, we performed in silico transcription factor perturbation using CellOracle based on the inferred NK GRNs. Briefly, the fitted GRN model was used to simulate PRDM1 KO by setting PRDM1 activity to zero and propagating the perturbation through the network (3 propagation steps). Transition probabilities were then estimated using a large kNN neighborhood (200 neighbors) and projected onto the UMAP embedding to calculate cell-identity shift vectors, which were visualized as vector fields on a grid. In parallel, a developmental reference flow was computed from pseudotime using a gradient-based approach and similarly represented as a grid-based vector field. To quantify the relationship between the perturbation-induced shift and the baseline developmental flow, we calculated a PS as the inner product between the 2 2D vectors at each grid location, capturing both directionality and magnitude; positive values indicate alignment with the developmental flow, whereas negative values indicate opposition. PSs were visualized on the NK UMAP and further summarized across pseudotime using both continuous representations and digitized pseudotime bins. The same workflow was repeated after restricting the analysis to cells within the aNK cluster to resolve PRDM1-dependent effects specifically along the aNK pseudotime continuum.

### scTenifoldKnk virtual KO and downstream analyses

To model PRDM1-dependent regulatory effects within aNK cells, we performed virtual KO using scTenifoldKnk on the aNK count matrix. aNK cells were subsetted from the NK object, and the count matrix was extracted for downstream network inference. For quality control and to reduce technical dominance from highly abundant housekeeping features, genes matching ribosomal, mitochondrial, and histone-related patterns were optionally removed prior to saving a filtered expression matrix for sensitivity analyses. Virtual KO was then performed by specifying PRDM1 as the perturbed gene and running the network construction using multiple subsampled cell sets and repeated network reconstructions (500 cells per subsample, 10 networks, 30 components, multi-core execution). The resulting differential regulation output was ranked by significance, and genes with strong perturbation effects were defined using a *Z*-score threshold (>2) while excluding PRDM1 itself. Perturbation effects were visualized using a volcano-style plot based on *Z*-scores and −log10(*P*) values with significant genes labeled.

Downstream functional interpretation was carried out using both over-representation and rank-based enrichment approaches. Over-representation analysis was performed on the significantly affected genes using GO-BP enrichment with multiple-testing correction (Benjamini–Hochberg) and stringent cutoffs (adjusted *P* < 0.01; *q* < 0.05). In parallel, GSEA was performed using the full gene list ranked by network *Z*-scores, including GO-BP GSEA and KEGG GSEA with standard gene set size constraints and a significance cutoff of *P* < 0.05, and representative enriched pathways were visualized using enrichment plots.

### Cytoscape subnetwork construction

To visualize a PRDM1-centered differential regulatory subnetwork, we extracted the inferred regulatory adjacency matrices from the wild-type and PRDM1-KO network reconstructions and computed the KO-minus-WT difference matrix. PRDM1 outgoing and incoming differential edges were ranked by absolute weight, and a neighborhood of top PRDM1-connected genes was selected to define a focused subnetwork. Differential edges within this subnetwork were then converted into an edge table containing source, target, signed weight, and absolute weight, and a node table was generated by integrating scTenifoldKnk gene-level statistics (*Z*-scores, *P* values, and significance calls) for the same set of nodes. The cleaned edge and node tables were exported as CSV files for downstream visualization and styling in Cytoscape software.

## Discussion

In this study, we reanalyzed a longitudinal cervical cancer scRNA-seq dataset collected before CRT and during treatment and observed a reproducible expansion of aNK cells together with induction of antiviral and cytotoxic transcriptional programs. These changes are consistent with the established ability of radiotherapy and chemotherapy to remodel the tumor immune microenvironment by promoting immunogenic cell death and DAMP release, which can enhance dendritic cell activation and antigen presentation, and by engaging inflammatory cytokine and interferon pathways that reshape immune-state composition. In parallel, irradiation can increase tumor cell immunogenicity through increased MHC class I antigen presentation and stress-associated changes that influence susceptibility to cytotoxic lymphocytes, including NK cells [6]. Together, these therapy-conditioned signals provide a plausible mechanistic context for the shift toward more differentiated or functionally primed NK states during on-treatment sampling in cervical cancer.

A key biological contribution of our work is the identification of PRDM1 as a CRT-associated candidate transcriptional regulator linked to the aNK compartment. Among the overlapping treatment-associated genes identified across onRT1 and onRT2, PRDM1 was prioritized for further investigation because it represented the strongest convergence of recurrent induction in our dataset, established immunological relevance, and consistency with our prior observations in tumor-infiltrating adaptive NK cells. PRDM1 (Blimp-1) is a well-established controller of lymphocyte differentiation programs and has been implicated in plasma cell maturation, effector differentiation, memory-related programs, and exhaustion-associated regulation in lymphocytes. In human NK cells, prior work has also shown that PRDM1 can regulate effector cytokine production and broader functional maturation [44]. In this context, the reappearance of PRDM1 among the shared CRT-associated genes made it a biologically compelling candidate regulator for deeper follow-up analysis, although we acknowledge that other overlapping genes may also contribute to CRT-associated NK cell remodeling and warrant future investigation. Consistent with this prioritization, PRDM1 remained prominent across multiple downstream analyses in the present study, including differential expression, trajectory-associated expression patterns, inferred regulatory network analyses, and in silico perturbation modeling. In addition, regulon-based analysis using pySCENIC provided orthogonal support that PRDM1 activity was enriched within the adaptive NK-associated transcriptional program. However, these network- and regulon-based findings remain inferential and should be interpreted as hypothesis-generating rather than definitive proof of a central regulatory role.

Our findings also align PRDM1 with the broader biology of immune memory and tumor-infiltrating adaptive-like NK states. Beyond the current cervical CRT setting, PRDM1 has been highlighted as a key transcriptional regulator in tumor-infiltrating aNK cells in ovarian cancer, supporting its potential cross-tumor relevance for aNK regulation [45]. In addition, recent tumor-immune surveillance work supports a functional requirement for Prdm1 in group 1 ILCs in cancer contexts [46], consistent with the idea that PRDM1 contributes to maintaining functional NK-state heterogeneity under tumor-associated stress.

Finally, our in silico perturbation analyses provide a predictive framework for interpreting the association of PRDM1 with CRT-enriched aNK states. CellOracle suggested that PRDM1 may influence inferred NK state-transition dynamics in a pseudotime-dependent manner, while scTenifoldKnk predicted broader downstream network consequences spanning immune-response modules and metabolism. Notably, oxidative phosphorylation and cellular respiration showed negative enrichment in GSEA, consistent with a predicted perturbation of mitochondrial respiratory programs following PRDM1 disruption. Together with STRING-based PPI clustering, these results support a hypothesis that PRDM1 may coordinate antiviral and inflammatory circuitry with cytotoxic and bioenergetic or proteostasis-associated programs in aNK cells. However, these findings remain computationally inferred and will require direct experimental validation to establish whether these predicted downstream effects occur in vivo.

There are important limitations to this study. First, mechanistic inferences are based on computational perturbation and network reconstruction and therefore require experimental validation. In addition, all analyses relied on a single public longitudinal scRNA-seq dataset, which, although valuable for examining treatment-associated adaptive NK cell remodeling, limits generalizability and requires validation in larger independent cohorts with more complete longitudinal sampling, richer clinicopathologic annotation, and complementary experimental approaches. The dataset also lacked sufficiently detailed and uniform clinical outcome information to robustly assess links between adaptive NK cell enrichment and treatment response or other patient-level correlates; thus, while the observed NK cell dynamics are biologically informative, their translational significance and interpatient consistency remain to be established. More broadly, recent work in computational oncology and gynecologic cancer research has emphasized that improving clinical prediction and biomarker discovery will likely require richer integration of molecular, pathological, and other multimodal data [47,48]. Gene-level differential expression was used as an exploratory screening step in sparse single-cell data, whereas pathway enrichment was interpreted more conservatively; moreover, because differential expression was performed at the cell level, within-patient dependence across repeated sampling time points was not explicitly modeled. Second, scRNA-seq captures transcriptional snapshots and does not directly measure protein activity, chromatin state, or posttranscriptional regulation, all of which are central to PRDM1 function. Third, treatment timing and patient heterogeneity, including HPV biology, potential CMV status, prior immune history, and regimen variability, can influence NK states but are incompletely captured in public datasets. Finally, although canonical adaptive NK features such as NKG2C up-regulation and reduced FcεRIγ expression are best established in peripheral blood HCMV-associated adaptive NK cells, these markers may not be uniformly preserved in tumor adaptive-like NK states; therefore, whether tumor-infiltrating aNK cells in cervical cancer represent canonical HCMV-associated aNK or convergent adaptive-like differentiation remains an open question.

Despite these limitations, our study links CRT-associated NK-state remodeling to a PRDM1-centered regulatory axis in aNK cells. Integrating longitudinal single-cell profiling with regulatory network inference and in silico perturbation suggests that PRDM1 is associated with aNK enrichment and may coordinate antiviral and cytotoxic transcriptional programs with mitochondrial respiratory pathways in the CRT-conditioned tumor microenvironment. Collectively, these results refine the transcriptional framework of therapy-driven NK adaptation in cervical cancer and highlight PRDM1 as a prominent candidate regulatory node within this response. Further experimental validation will be required to determine whether PRDM1 directly regulates adaptive NK cell enrichment and functional remodeling during CRT.

## Ethical Approval

Not applicable. This study used publicly available, de-identified data deposited in the NCBI GEO under accession GSE297041. The original data collection and data generation were approved by the relevant institutional ethics committee(s) and performed with informed consent, in accordance with the Declaration of Helsinki. The authors had no access to identifiable participant information.

## Data Availability

All code used for data processing, analysis, and figure generation in this study is publicly available at https://github.com/yizhesuncode/Cervical_RT_aNK_codes/.
