# Supplementary material for: PRDM1 Is Associated with Chemoradiotherapy-Associated Enrichment of Adaptive NK Cells in Cervical Cancer
Source: Comput Struct Biotechnol J. 2026 May 7;35(1):0092. doi: 10.34133/csbj.0092 (PMC13150072; doi:10.34133/csbj.0092)
Supplement: Supplementary 1 — Figs. S1 to S3 Table S1 [file csbj.0092.f1.zip › Supplementary Table 1.pdf]

Supplementary Table 1. Gene signatures used for aNK subcluster annotation

CellType	tissueType	cellName	geneSymbolmore1(Positively expressed)	geneSymbolmore2 (Negatively expressed)
0	Immune system	Adaptive_NK	FCGR3A	KLRC1,FCER1G,SYK
0	Immune system	Adaptive_NK	FGFBP2	KLRC1,FCER1G,SYK
0	Immune system	Adaptive_NK	KLRF1	KLRC1,FCER1G,SYK
0	Immune system	Adaptive_NK	KLF2	KLRC1,FCER1G,SYK
0	Immune system	Adaptive_NK	PLAC8	KLRC1,FCER1G,SYK
0	Immune system	Adaptive_NK	GZMB	KLRC1,FCER1G,SYK
0	Immune system	Adaptive_NK	GZMH	KLRC1,FCER1G,SYK
0	Immune system	Adaptive_NK	CX3CR1	KLRC1,FCER1G,SYK
0	Immune system	Adaptive_NK	ARL4C	KLRC1,FCER1G,SYK
0	Immune system	Adaptive_NK	PRF1	KLRC1,FCER1G,SYK
0	Immune system	Adaptive_NK	EFHD2	KLRC1,FCER1G,SYK
0	Immune system	Adaptive_NK	CYBA	KLRC1,FCER1G,SYK
0	Immune system	Adaptive_NK	NKG7	KLRC1,FCER1G,SYK
0	Immune system	Adaptive_NK	PLEK	KLRC1,FCER1G,SYK
0	Immune system	Adaptive_NK	S1PR5	KLRC1,FCER1G,SYK
0	Immune system	Adaptive_NK	CST7	KLRC1,FCER1G,SYK
0	Immune system	Adaptive_NK	ZEB2	KLRC1,FCER1G,SYK
0	Immune system	Adaptive_NK	DTHD1	KLRC1,FCER1G,SYK
0	Immune system	Adaptive_NK	SPON2	KLRC1,FCER1G,SYK
0	Immune system	Adaptive_NK	ADGRG1	KLRC1,FCER1G,SYK
0	Immune system	Adaptive_NK	RIPOR2	KLRC1,FCER1G,SYK
0	Immune system	Adaptive_NK	S100A4	KLRC1,FCER1G,SYK
0	Immune system	Adaptive_NK	TGFBR3	KLRC1,FCER1G,SYK
0	Immune system	Adaptive_NK	PRSS23	KLRC1,FCER1G,SYK
0	Immune system	Adaptive_NK	IGFBP7	KLRC1,FCER1G,SYK
0	Immune system	Adaptive_NK	GZMM	KLRC1,FCER1G,SYK
0	Immune system	Adaptive_NK	TTC38	KLRC1,FCER1G,SYK
0	Immune system	Adaptive_NK	PFN1	KLRC1,FCER1G,SYK
0	Immune system	Adaptive_NK	CD247	KLRC1,FCER1G,SYK
0	Immune system	Adaptive_NK	CEP78	KLRC1,FCER1G,SYK
0	Immune system	Adaptive_NK	LITAF	KLRC1,FCER1G,SYK
0	Immune system	Adaptive_NK	ACTB	KLRC1,FCER1G,SYK
0	Immune system	Adaptive_NK	RAP1B	KLRC1,FCER1G,SYK
0	Immune system	Adaptive_NK	FLNA	KLRC1,FCER1G,SYK
0	Immune system	Adaptive_NK	EMP3	KLRC1,FCER1G,SYK
0	Immune system	Adaptive_NK	B2M	KLRC1,FCER1G,SYK
0	Immune system	Adaptive_NK	C12orf75	KLRC1,FCER1G,SYK
0	Immune system	Adaptive_NK	CALM1	KLRC1,FCER1G,SYK
0	Immune system	Adaptive_NK	KLRD1	KLRC1,FCER1G,SYK
0	Immune system	Adaptive_NK	GNG2	KLRC1,FCER1G,SYK
0	Immune system	Adaptive_NK	TRBC1	KLRC1,FCER1G,SYK
0	Immune system	Adaptive_NK	CCL4L2	KLRC1,FCER1G,SYK
0	Immune system	Adaptive_NK	HLA-C	KLRC1,FCER1G,SYK
0	Immune system	Adaptive_NK	ACTG1	KLRC1,FCER1G,SYK
0	Immune system	Adaptive_NK	ANXA1	KLRC1,FCER1G,SYK
0	Immune system	Adaptive_NK	C1orf21	KLRC1,FCER1G,SYK
0	Immune system	Adaptive_NK	SORL1	KLRC1,FCER1G,SYK
0	Immune system	Adaptive_NK	HLA-E	KLRC1,FCER1G,SYK
0	Immune system	Adaptive_NK	AHNAK	KLRC1,FCER1G,SYK
0	Immune system	Adaptive_NK	HLA-B	KLRC1,FCER1G,SYK
0	Immune system	Adaptive_NK	ABHD17A	KLRC1,FCER1G,SYK
0	Immune system	Adaptive_NK	FGL2	KLRC1,FCER1G,SYK
0	Immune system	Adaptive_NK	PPP2R5C	KLRC1,FCER1G,SYK
0	Immune system	Adaptive_NK	COL6A2	KLRC1,FCER1G,SYK

0	Immune system	Adaptive_NK	TSC22D3	KLRC1,FCER1G,SYK
0	Immune system	Adaptive_NK	S100A6	KLRC1,FCER1G,SYK
0	Immune system	Adaptive_NK	LYAR	KLRC1,FCER1G,SYK
0	Immune system	Adaptive_NK	GK5	KLRC1,FCER1G,SYK
0	Immune system	Adaptive_NK	TPST2	KLRC1,FCER1G,SYK
0	Immune system	Adaptive_NK	LPCAT1	KLRC1,FCER1G,SYK
0	Immune system	Adaptive_NK	KIR2DL3	KLRC1,FCER1G,SYK
0	Immune system	Adaptive_NK	CFL1	KLRC1,FCER1G,SYK
0	Immune system	Adaptive_NK	SH3BP5	KLRC1,FCER1G,SYK
0	Immune system	Adaptive_NK	XBP1	KLRC1,FCER1G,SYK
0	Immune system	Adaptive_NK	ITGB2	KLRC1,FCER1G,SYK
0	Immune system	Adaptive_NK	FCRL6	KLRC1,FCER1G,SYK
0	Immune system	Adaptive_NK	PRKCB	KLRC1,FCER1G,SYK
0	Immune system	Adaptive_NK	MYL12A	KLRC1,FCER1G,SYK
0	Immune system	Adaptive_NK	HLA-DQA2	KLRC1,FCER1G,SYK
0	Immune system	Adaptive_NK	MYBL1	KLRC1,FCER1G,SYK
0	Immune system	Adaptive_NK	RAB29	KLRC1,FCER1G,SYK
0	Immune system	Adaptive_NK	DSTN	KLRC1,FCER1G,SYK
0	Immune system	Adaptive_NK	PTGDR	KLRC1,FCER1G,SYK
0	Immune system	Adaptive_NK	RASGRP2	KLRC1,FCER1G,SYK
0	Immune system	Adaptive_NK	MBP	KLRC1,FCER1G,SYK
0	Immune system	Adaptive_NK	F2R	KLRC1,FCER1G,SYK
0	Immune system	Adaptive_NK	ARPC2	KLRC1,FCER1G,SYK
0	Immune system	Adaptive_NK	SYNE2	KLRC1,FCER1G,SYK
0	Immune system	Adaptive_NK	LAIR1	KLRC1,FCER1G,SYK
0	Immune system	Adaptive_NK	CCL4	KLRC1,FCER1G,SYK
0	Immune system	Adaptive_NK	CD47	KLRC1,FCER1G,SYK
0	Immune system	Adaptive_NK	TBX21	KLRC1,FCER1G,SYK
0	Immune system	Adaptive_NK	CMC1	KLRC1,FCER1G,SYK
0	Immune system	Adaptive_NK	ANXA4	KLRC1,FCER1G,SYK
0	Immune system	Adaptive_NK	RGS9	KLRC1,FCER1G,SYK
0	Immune system	Adaptive_NK	TMSB10	KLRC1,FCER1G,SYK
0	Immune system	Adaptive_NK	PRDM1	KLRC1,FCER1G,SYK
0	Immune system	Adaptive_NK	RASA3	KLRC1,FCER1G,SYK
0	Immune system	Adaptive_NK	CD53	KLRC1,FCER1G,SYK
0	Immune system	Adaptive_NK	SPN	KLRC1,FCER1G,SYK
0	Immune system	Adaptive_NK	ADRB2	KLRC1,FCER1G,SYK
0	Immune system	Adaptive_NK	KLF3	KLRC1,FCER1G,SYK
0	Immune system	Adaptive_NK	ADD3	KLRC1,FCER1G,SYK
0	Immune system	Adaptive_NK	HLA-F	KLRC1,FCER1G,SYK
0	Immune system	Adaptive_NK	TFDP2	KLRC1,FCER1G,SYK
0	Immune system	Adaptive_NK	SSBP3	KLRC1,FCER1G,SYK
0	Immune system	Adaptive_NK	SYNE1	KLRC1,FCER1G,SYK
0	Immune system	Adaptive_NK	SYTL3	KLRC1,FCER1G,SYK
0	Immune system	Adaptive_NK	GLRX	KLRC1,FCER1G,SYK
0	Immune system	Adaptive_NK	HLA-DPB1	KLRC1,FCER1G,SYK
0	Immune system	Adaptive_NK	RAP2B	KLRC1,FCER1G,SYK
0	Immune system	Adaptive_NK	UCP2	KLRC1,FCER1G,SYK
0	Immune system	Adaptive_NK	TXNIP	KLRC1,FCER1G,SYK
0	Immune system	Adaptive_NK	VCL	KLRC1,FCER1G,SYK
0	Immune system	Adaptive_NK	MGAT4A	KLRC1,FCER1G,SYK
0	Immune system	Adaptive_NK	GZMA	KLRC1,FCER1G,SYK
0	Immune system	Adaptive_NK	GTF3C1	KLRC1,FCER1G,SYK
0	Immune system	Adaptive_NK	PTGER2	KLRC1,FCER1G,SYK
0	Immune system	Adaptive_NK	PTPRC	KLRC1,FCER1G,SYK
0	Immune system	Adaptive_NK	BIN2	KLRC1,FCER1G,SYK
0	Immune system	Adaptive_NK	GIMAP4	KLRC1,FCER1G,SYK
0	Immune system	Adaptive_NK	PXN	KLRC1,FCER1G,SYK

0	Immune system	Adaptive_NK	ABI3	KLRC1,FCER1G,SYK
0	Immune system	Adaptive_NK	ADGRE5	KLRC1,FCER1G,SYK
0	Immune system	Adaptive_NK	SRGN	KLRC1,FCER1G,SYK
0	Immune system	Adaptive_NK	HDDC2	KLRC1,FCER1G,SYK
0	Immune system	Adaptive_NK	ICAM2	KLRC1,FCER1G,SYK
0	Immune system	Adaptive_NK	TPM4	KLRC1,FCER1G,SYK
0	Immune system	Adaptive_NK	KLRC2	KLRC1,FCER1G,SYK
0	Immune system	Adaptive_NK	CAP1	KLRC1,FCER1G,SYK
0	Immune system	Adaptive_NK	LINC00861	KLRC1,FCER1G,SYK
0	Immune system	Adaptive_NK	GRAP2	KLRC1,FCER1G,SYK
0	Immune system	Adaptive_NK	EIF1	KLRC1,FCER1G,SYK
0	Immune system	Adaptive_NK	IFNG	KLRC1,FCER1G,SYK
0	Immune system	Adaptive_NK	ITGAL	KLRC1,FCER1G,SYK
0	Immune system	Adaptive_NK	PTPRE	KLRC1,FCER1G,SYK
0	Immune system	Adaptive_NK	PTGER4	KLRC1,FCER1G,SYK
0	Immune system	Adaptive_NK	HLA-DPA1	KLRC1,FCER1G,SYK
0	Immune system	Adaptive_NK	CDC42SE1	KLRC1,FCER1G,SYK
0	Immune system	Adaptive_NK	MAF	KLRC1,FCER1G,SYK
0	Immune system	Adaptive_NK	CTBP2	KLRC1,FCER1G,SYK
0	Immune system	Adaptive_NK	CDKN2D	KLRC1,FCER1G,SYK
0	Immune system	Adaptive_NK	CYTH1	KLRC1,FCER1G,SYK
0	Immune system	Adaptive_NK	MYO1G	KLRC1,FCER1G,SYK
0	Immune system	Adaptive_NK	SH3KBP1	KLRC1,FCER1G,SYK
0	Immune system	Adaptive_NK	YWHAB	KLRC1,FCER1G,SYK
0	Immune system	Adaptive_NK	CDC25B	KLRC1,FCER1G,SYK
0	Immune system	Adaptive_NK	ATM	KLRC1,FCER1G,SYK
0	Immune system	Adaptive_NK	ACTR3	KLRC1,FCER1G,SYK
0	Immune system	Adaptive_NK	RAB9A	KLRC1,FCER1G,SYK
0	Immune system	Adaptive_NK	RORA	KLRC1,FCER1G,SYK
0	Immune system	Adaptive_NK	GAB3	KLRC1,FCER1G,SYK
0	Immune system	Adaptive_NK	KLF13	KLRC1,FCER1G,SYK
0	Immune system	Adaptive_NK	KLF6	KLRC1,FCER1G,SYK
0	Immune system	Adaptive_NK	CAST	KLRC1,FCER1G,SYK
0	Immune system	Adaptive_NK	IQGAP2	KLRC1,FCER1G,SYK
0	Immune system	Adaptive_NK	RPL3	KLRC1,FCER1G,SYK
0	Immune system	Adaptive_NK	CD320	KLRC1,FCER1G,SYK
0	Immune system	Adaptive_NK	IGF2R	KLRC1,FCER1G,SYK
0	Immune system	Adaptive_NK	IFITM1	KLRC1,FCER1G,SYK
0	Immune system	Adaptive_NK	GPSM3	KLRC1,FCER1G,SYK
0	Immune system	Adaptive_NK	FTL	KLRC1,FCER1G,SYK
0	Immune system	Adaptive_NK	RHOG	KLRC1,FCER1G,SYK
0	Immune system	Adaptive_NK	CD3E	KLRC1,FCER1G,SYK
0	Immune system	Adaptive_NK	EBP	KLRC1,FCER1G,SYK
0	Immune system	Adaptive_NK	VASP	KLRC1,FCER1G,SYK
0	Immune system	Adaptive_NK	ARID5B	KLRC1,FCER1G,SYK
0	Immune system	Adaptive_NK	SH2D2A	KLRC1,FCER1G,SYK
0	Immune system	Adaptive_NK	MIAT	KLRC1,FCER1G,SYK
0	Immune system	Adaptive_NK	PTP4A2	KLRC1,FCER1G,SYK
0	Immune system	Adaptive_NK	HSPA8	KLRC1,FCER1G,SYK
0	Immune system	Adaptive_NK	IFITM2	KLRC1,FCER1G,SYK
0	Immune system	Adaptive_NK	PYHIN1	KLRC1,FCER1G,SYK
0	Immune system	Adaptive_NK	NDUFB2	KLRC1,FCER1G,SYK
0	Immune system	Adaptive_NK	RPA2	KLRC1,FCER1G,SYK
0	Immune system	Adaptive_NK	HIPK2	KLRC1,FCER1G,SYK
0	Immune system	Adaptive_NK	SIGIRR	KLRC1,FCER1G,SYK
0	Immune system	Adaptive_NK	EIF4G3	KLRC1,FCER1G,SYK
0	Immune system	Adaptive_NK	NDUFB7	KLRC1,FCER1G,SYK
0	Immune system	Adaptive_NK	APMAP	KLRC1,FCER1G,SYK

0	Immune system	Adaptive_NK	CD300A	KLRC1,FCER1G,SYK
0	Immune system	Adaptive_NK	CTSC	KLRC1,FCER1G,SYK
0	Immune system	Adaptive_NK	CCDC88C	KLRC1,FCER1G,SYK
0	Immune system	Adaptive_NK	PDIA3	KLRC1,FCER1G,SYK
0	Immune system	Adaptive_NK	STK10	KLRC1,FCER1G,SYK
0	Immune system	Adaptive_NK	TOB1	KLRC1,FCER1G,SYK
0	Immune system	Adaptive_NK	YWHAZ	KLRC1,FCER1G,SYK
0	Immune system	Adaptive_NK	PPIB	KLRC1,FCER1G,SYK
0	Immune system	Adaptive_NK	METRNL	KLRC1,FCER1G,SYK
0	Immune system	Adaptive_NK	HLA-DRB1	KLRC1,FCER1G,SYK
0	Immune system	Adaptive_NK	FAM49B	KLRC1,FCER1G,SYK
0	Immune system	Adaptive_NK	ARPC5	KLRC1,FCER1G,SYK
0	Immune system	Adaptive_NK	MYADM	KLRC1,FCER1G,SYK
0	Immune system	Adaptive_NK	TERF1	KLRC1,FCER1G,SYK
0	Immune system	Adaptive_NK	ARHGDIB	KLRC1,FCER1G,SYK
0	Immune system	Adaptive_NK	PPP1CA	KLRC1,FCER1G,SYK
0	Immune system	Adaptive_NK	PTPN12	KLRC1,FCER1G,SYK
0	Immune system	Adaptive_NK	VAV3	KLRC1,FCER1G,SYK
0	Immune system	Adaptive_NK	RTN4	KLRC1,FCER1G,SYK
0	Immune system	Adaptive_NK	WDR1	KLRC1,FCER1G,SYK
0	Immune system	Adaptive_NK	TLE4	KLRC1,FCER1G,SYK
0	Immune system	Adaptive_NK	P4HB	KLRC1,FCER1G,SYK
0	Immune system	Adaptive_NK	ZBTB38	KLRC1,FCER1G,SYK
0	Immune system	Adaptive_NK	CLIC3	KLRC1,FCER1G,SYK
0	Immune system	Adaptive_NK	RGS19	KLRC1,FCER1G,SYK
0	Immune system	Adaptive_NK	USP28	KLRC1,FCER1G,SYK
0	Immune system	Adaptive_NK	FGR	KLRC1,FCER1G,SYK
0	Immune system	Adaptive_NK	TNFRSF1B	KLRC1,FCER1G,SYK
0	Immune system	Adaptive_NK	RPL21	KLRC1,FCER1G,SYK
0	Immune system	Adaptive_NK	OSTF1	KLRC1,FCER1G,SYK
0	Immune system	Adaptive_NK	PRR5L	KLRC1,FCER1G,SYK
0	Immune system	Adaptive_NK	HSPA5	KLRC1,FCER1G,SYK
0	Immune system	Adaptive_NK	TMBIM6	KLRC1,FCER1G,SYK
0	Immune system	Adaptive_NK	DBI	KLRC1,FCER1G,SYK
0	Immune system	Adaptive_NK	TMEM181	KLRC1,FCER1G,SYK
0	Immune system	Adaptive_NK	ACTR2	KLRC1,FCER1G,SYK
0	Immune system	Adaptive_NK	CORO1A	KLRC1,FCER1G,SYK
0	Immune system	Adaptive_NK	RAC2	KLRC1,FCER1G,SYK
0	Immune system	Adaptive_NK	ARHGAP25	KLRC1,FCER1G,SYK
0	Immune system	Adaptive_NK	UPP1	KLRC1,FCER1G,SYK
0	Immune system	Adaptive_NK	LYN	KLRC1,FCER1G,SYK
0	Immune system	Adaptive_NK	TMEM173	KLRC1,FCER1G,SYK
0	Immune system	Adaptive_NK	AES	KLRC1,FCER1G,SYK
0	Immune system	Adaptive_NK	MYO1F	KLRC1,FCER1G,SYK
0	Immune system	Adaptive_NK	SASH3	KLRC1,FCER1G,SYK
0	Immune system	Adaptive_NK	BATF	KLRC1,FCER1G,SYK
0	Immune system	Adaptive_NK	GIMAP1	KLRC1,FCER1G,SYK
0	Immune system	Adaptive_NK	UBE2F	KLRC1,FCER1G,SYK
0	Immune system	Adaptive_NK	CAPN2	KLRC1,FCER1G,SYK
0	Immune system	Adaptive_NK	SLC9A3R1	KLRC1,FCER1G,SYK
0	Immune system	Adaptive_NK	SPCS3	KLRC1,FCER1G,SYK
0	Immune system	Adaptive_NK	GMFG	KLRC1,FCER1G,SYK
0	Immune system	Adaptive_NK	SMAP2	KLRC1,FCER1G,SYK
0	Immune system	Adaptive_NK	HNRNPF	KLRC1,FCER1G,SYK
0	Immune system	Adaptive_NK	CD99	KLRC1,FCER1G,SYK
0	Immune system	Adaptive_NK	CDC42	KLRC1,FCER1G,SYK
0	Immune system	Adaptive_NK	RHOA	KLRC1,FCER1G,SYK
0	Immune system	Adaptive_NK	ARPC4	KLRC1,FCER1G,SYK

0	Immune system	Adaptive_NK	BIN1	KLRC1,FCER1G,SYK
0	Immune system	Adaptive_NK	ORAI1	KLRC1,FCER1G,SYK
0	Immune system	Adaptive_NK	DHRS7	KLRC1,FCER1G,SYK
0	Immune system	Adaptive_NK	ANXA2	KLRC1,FCER1G,SYK
0	Immune system	Adaptive_NK	TPP1	KLRC1,FCER1G,SYK
0	Immune system	Adaptive_NK	ATP2B4	KLRC1,FCER1G,SYK
0	Immune system	Adaptive_NK	CDC42EP3	KLRC1,FCER1G,SYK
0	Immune system	Adaptive_NK	UTRN	KLRC1,FCER1G,SYK
0	Immune system	Adaptive_NK	GNAI2	KLRC1,FCER1G,SYK
0	Immune system	Adaptive_NK	FYN	KLRC1,FCER1G,SYK
0	Immune system	Adaptive_NK	TES	KLRC1,FCER1G,SYK
0	Immune system	Adaptive_NK	SDF2L1	KLRC1,FCER1G,SYK
0	Immune system	Adaptive_NK	TGFBR1	KLRC1,FCER1G,SYK
0	Immune system	Adaptive_NK	RNF19A	KLRC1,FCER1G,SYK
0	Immune system	Adaptive_NK	KLRG1	KLRC1,FCER1G,SYK
0	Immune system	Adaptive_NK	PARP15	KLRC1,FCER1G,SYK
0	Immune system	Adaptive_NK	MED15	KLRC1,FCER1G,SYK
0	Immune system	Adaptive_NK	SLC15A4	KLRC1,FCER1G,SYK
0	Immune system	Adaptive_NK	S1PR4	KLRC1,FCER1G,SYK
0	Immune system	Adaptive_NK	LLGL2	KLRC1,FCER1G,SYK
0	Immune system	Adaptive_NK	CALR	KLRC1,FCER1G,SYK
0	Immune system	Adaptive_NK	SFT2D1	KLRC1,FCER1G,SYK
0	Immune system	Adaptive_NK	MIEN1	KLRC1,FCER1G,SYK
0	Immune system	Adaptive_NK	TUBA4A	KLRC1,FCER1G,SYK
0	Immune system	Adaptive_NK	NCOA1	KLRC1,FCER1G,SYK
0	Immune system	Adaptive_NK	SUN2	KLRC1,FCER1G,SYK
0	Immune system	Adaptive_NK	UHMK1	KLRC1,FCER1G,SYK
0	Immune system	Adaptive_NK	ANXA6	KLRC1,FCER1G,SYK
0	Immune system	Adaptive_NK	MIDN	KLRC1,FCER1G,SYK
0	Immune system	Adaptive_NK	TNFRSF14	KLRC1,FCER1G,SYK
0	Immune system	Adaptive_NK	PTPN18	KLRC1,FCER1G,SYK
0	Immune system	Adaptive_NK	GPR65	KLRC1,FCER1G,SYK
0	Immune system	Adaptive_NK	AKNA	KLRC1,FCER1G,SYK
0	Immune system	Adaptive_NK	MANF	KLRC1,FCER1G,SYK
0	Immune system	Adaptive_NK	LCK	KLRC1,FCER1G,SYK
0	Immune system	Adaptive_NK	LMAN2	KLRC1,FCER1G,SYK
0	Immune system	Adaptive_NK	MOB3A	KLRC1,FCER1G,SYK
0	Immune system	Adaptive_NK	PRELID1	KLRC1,FCER1G,SYK
0	Immune system	Adaptive_NK	CAPZB	KLRC1,FCER1G,SYK
0	Immune system	Adaptive_NK	CREM	KLRC1,FCER1G,SYK
0	Immune system	Adaptive_NK	FBXW5	KLRC1,FCER1G,SYK
0	Immune system	Adaptive_NK	CDK2AP2	KLRC1,FCER1G,SYK
0	Immune system	Adaptive_NK	SH3BP2	KLRC1,FCER1G,SYK
0	Immune system	Adaptive_NK	MSN	KLRC1,FCER1G,SYK
0	Immune system	Adaptive_NK	PPIA	KLRC1,FCER1G,SYK
0	Immune system	Adaptive_NK	YWHAQ	KLRC1,FCER1G,SYK
0	Immune system	Adaptive_NK	PRMT2	KLRC1,FCER1G,SYK
0	Immune system	Adaptive_NK	ATP1A1	KLRC1,FCER1G,SYK
0	Immune system	Adaptive_NK	CLIC1	KLRC1,FCER1G,SYK
0	Immune system	Adaptive_NK	STK38	KLRC1,FCER1G,SYK
0	Immune system	Adaptive_NK	DIAPH1	KLRC1,FCER1G,SYK
0	Immune system	Adaptive_NK	DENND2D	KLRC1,FCER1G,SYK
0	Immune system	Adaptive_NK	S100A10	KLRC1,FCER1G,SYK
0	Immune system	Adaptive_NK	SUPT4H1	KLRC1,FCER1G,SYK
0	Immune system	Adaptive_NK	ATP2B1-AS1	KLRC1,FCER1G,SYK
0	Immune system	Adaptive_NK	SYNGR1	KLRC1,FCER1G,SYK
0	Immune system	Adaptive_NK	MAPRE2	KLRC1,FCER1G,SYK
0	Immune system	Adaptive_NK	HSP90B1	KLRC1,FCER1G,SYK

0	Immune system	Adaptive_NK	G6PD	KLRC1,FCER1G,SYK
0	Immune system	Adaptive_NK	TBCB	KLRC1,FCER1G,SYK
0	Immune system	Adaptive_NK	TRPV2	KLRC1,FCER1G,SYK
0	Immune system	Adaptive_NK	GRK6	KLRC1,FCER1G,SYK
0	Immune system	Adaptive_NK	TIMP1	KLRC1,FCER1G,SYK
0	Immune system	Adaptive_NK	MTPN	KLRC1,FCER1G,SYK
0	Immune system	Adaptive_NK	LDLR	KLRC1,FCER1G,SYK
0	Immune system	Adaptive_NK	ZAP70	KLRC1,FCER1G,SYK
0	Immune system	Adaptive_NK	C12orf57	KLRC1,FCER1G,SYK
0	Immune system	Adaptive_NK	CISD3	KLRC1,FCER1G,SYK
0	Immune system	Adaptive_NK	RNF167	KLRC1,FCER1G,SYK
0	Immune system	Adaptive_NK	IVNS1ABP	KLRC1,FCER1G,SYK
0	Immune system	Adaptive_NK	ARL6IP1	KLRC1,FCER1G,SYK
0	Immune system	Adaptive_NK	PPP1R18	KLRC1,FCER1G,SYK
0	Immune system	Adaptive_NK	TPM3	KLRC1,FCER1G,SYK
0	Immune system	Adaptive_NK	GIMAP7	KLRC1,FCER1G,SYK
0	Immune system	Adaptive_NK	SNF8	KLRC1,FCER1G,SYK
0	Immune system	Adaptive_NK	SEC11C	KLRC1,FCER1G,SYK
0	Immune system	Adaptive_NK	LBR	KLRC1,FCER1G,SYK
0	Immune system	Adaptive_NK	RAB8A	KLRC1,FCER1G,SYK
0	Immune system	Adaptive_NK	ZNF276	KLRC1,FCER1G,SYK
0	Immune system	Adaptive_NK	PIP4K2A	KLRC1,FCER1G,SYK
0	Immune system	Adaptive_NK	RPS26	KLRC1,FCER1G,SYK
0	Immune system	Adaptive_NK	RASSF1	KLRC1,FCER1G,SYK
0	Immune system	Adaptive_NK	BCL11B	KLRC1,FCER1G,SYK
0	Immune system	Adaptive_NK	PCBP1	KLRC1,FCER1G,SYK
0	Immune system	Adaptive_NK	FKBP11	KLRC1,FCER1G,SYK
0	Immune system	Adaptive_NK	TMEM50A	KLRC1,FCER1G,SYK
0	Immune system	Adaptive_NK	MYDGF	KLRC1,FCER1G,SYK
0	Immune system	Adaptive_NK	RNF126	KLRC1,FCER1G,SYK
0	Immune system	Adaptive_NK	RRBP1	KLRC1,FCER1G,SYK
0	Immune system	Adaptive_NK	PLEKHA1	KLRC1,FCER1G,SYK
0	Immune system	Adaptive_NK	SELPLG	KLRC1,FCER1G,SYK
0	Immune system	Adaptive_NK	SH2D1B	KLRC1,FCER1G,SYK
0	Immune system	Adaptive_NK	RAB5C	KLRC1,FCER1G,SYK
0	Immune system	Adaptive_NK	ARRDC3	KLRC1,FCER1G,SYK
0	Immune system	Adaptive_NK	ARL6IP5	KLRC1,FCER1G,SYK
0	Immune system	Adaptive_NK	TMEM2	KLRC1,FCER1G,SYK
0	Immune system	Adaptive_NK	CCDC85B	KLRC1,FCER1G,SYK
0	Immune system	Adaptive_NK	ARPC1B	KLRC1,FCER1G,SYK
0	Immune system	Adaptive_NK	SIRT2	KLRC1,FCER1G,SYK
0	Immune system	Adaptive_NK	CD226	KLRC1,FCER1G,SYK
0	Immune system	Adaptive_NK	LRRFIP1	KLRC1,FCER1G,SYK
0	Immune system	Adaptive_NK	RAB10	KLRC1,FCER1G,SYK
0	Immune system	Adaptive_NK	ATP1B3	KLRC1,FCER1G,SYK
0	Immune system	Adaptive_NK	PDIA6	KLRC1,FCER1G,SYK
0	Immune system	Adaptive_NK	SRPK2	KLRC1,FCER1G,SYK
0	Immune system	Adaptive_NK	IQGAP1	KLRC1,FCER1G,SYK
0	Immune system	Adaptive_NK	PGAM1	KLRC1,FCER1G,SYK
0	Immune system	Adaptive_NK	CAPNS1	KLRC1,FCER1G,SYK
0	Immune system	Adaptive_NK	TMED9	KLRC1,FCER1G,SYK
0	Immune system	Adaptive_NK	MFSD10	KLRC1,FCER1G,SYK
0	Immune system	Adaptive_NK	ATP5F1	KLRC1,FCER1G,SYK
0	Immune system	Adaptive_NK	DECR1	KLRC1,FCER1G,SYK
0	Immune system	Adaptive_NK	PDLIM2	KLRC1,FCER1G,SYK
0	Immune system	Adaptive_NK	SERPINB1	KLRC1,FCER1G,SYK
0	Immune system	Adaptive_NK	CCDC82	KLRC1,FCER1G,SYK
0	Immune system	Adaptive_NK	CEBPB	KLRC1,FCER1G,SYK

0	Immune system	Adaptive_NK	ITGB1BP1	KLRC1,FCER1G,SYK
0	Immune system	Adaptive_NK	M6PR	KLRC1,FCER1G,SYK
0	Immune system	Adaptive_NK	TMEM59	KLRC1,FCER1G,SYK
0	Immune system	Adaptive_NK	RECQL	KLRC1,FCER1G,SYK
0	Immune system	Adaptive_NK	RGS14	KLRC1,FCER1G,SYK
0	Immune system	Adaptive_NK	PLEKHF1	KLRC1,FCER1G,SYK
0	Immune system	Adaptive_NK	NFATC2	KLRC1,FCER1G,SYK
0	Immune system	Adaptive_NK	PPP2R5A	KLRC1,FCER1G,SYK
0	Immune system	Adaptive_NK	SEC61B	KLRC1,FCER1G,SYK
0	Immune system	Adaptive_NK	SYTL1	KLRC1,FCER1G,SYK
0	Immune system	Adaptive_NK	SUB1	KLRC1,FCER1G,SYK
0	Immune system	Adaptive_NK	TLN1	KLRC1,FCER1G,SYK
0	Immune system	Adaptive_NK	SPCS2	KLRC1,FCER1G,SYK
0	Immune system	Adaptive_NK	PDAP1	KLRC1,FCER1G,SYK
0	Immune system	Adaptive_NK	VAMP2	KLRC1,FCER1G,SYK
0	Immune system	Adaptive_NK	SLAMF7	KLRC1,FCER1G,SYK
0	Immune system	Adaptive_NK	CRBN	KLRC1,FCER1G,SYK
0	Immune system	Adaptive_NK	SELENOT	KLRC1,FCER1G,SYK
0	Immune system	Adaptive_NK	TECR	KLRC1,FCER1G,SYK
0	Immune system	Adaptive_NK	RNF149	KLRC1,FCER1G,SYK
0	Immune system	Adaptive_NK	SIPA1	KLRC1,FCER1G,SYK
0	Immune system	Adaptive_NK	EIF4EBP2	KLRC1,FCER1G,SYK
0	Immune system	Adaptive_NK	HSH2D	KLRC1,FCER1G,SYK
0	Immune system	Adaptive_NK	IMP3	KLRC1,FCER1G,SYK
0	Immune system	Adaptive_NK	CIB1	KLRC1,FCER1G,SYK
0	Immune system	Adaptive_NK	LRP10	KLRC1,FCER1G,SYK
0	Immune system	Adaptive_NK	ARPC5L	KLRC1,FCER1G,SYK
0	Immune system	Adaptive_NK	RAPGEF1	KLRC1,FCER1G,SYK
0	Immune system	Adaptive_NK	POLR3GL	KLRC1,FCER1G,SYK
0	Immune system	Adaptive_NK	RNF169	KLRC1,FCER1G,SYK
0	Immune system	Adaptive_NK	ZFAND6	KLRC1,FCER1G,SYK
0	Immune system	Adaptive_NK	ARHGAP30	KLRC1,FCER1G,SYK
0	Immune system	Adaptive_NK	STOM	KLRC1,FCER1G,SYK
0	Immune system	Adaptive_NK	TCF25	KLRC1,FCER1G,SYK
0	Immune system	Adaptive_NK	CTDSP1	KLRC1,FCER1G,SYK
0	Immune system	Adaptive_NK	PSMB10	KLRC1,FCER1G,SYK
0	Immune system	Adaptive_NK	ATOX1	KLRC1,FCER1G,SYK
0	Immune system	Adaptive_NK	SYAP1	KLRC1,FCER1G,SYK
0	Immune system	Adaptive_NK	CYB561D2	KLRC1,FCER1G,SYK
0	Immune system	Adaptive_NK	EIF4B	KLRC1,FCER1G,SYK
0	Immune system	Adaptive_NK	TAF7	KLRC1,FCER1G,SYK
0	Immune system	Adaptive_NK	SRPRA	KLRC1,FCER1G,SYK
0	Immune system	Adaptive_NK	CANX	KLRC1,FCER1G,SYK
0	Immune system	Adaptive_NK	RNASEH2C	KLRC1,FCER1G,SYK
0	Immune system	Adaptive_NK	IDI1	KLRC1,FCER1G,SYK
0	Immune system	Adaptive_NK	SSBP4	KLRC1,FCER1G,SYK
0	Immune system	Adaptive_NK	C16orf54	KLRC1,FCER1G,SYK
0	Immune system	Adaptive_NK	CYFIP2	KLRC1,FCER1G,SYK
0	Immune system	Adaptive_NK	RNPEPL1	KLRC1,FCER1G,SYK
0	Immune system	Adaptive_NK	ARHGDIA	KLRC1,FCER1G,SYK
0	Immune system	Adaptive_NK	CELF2	KLRC1,FCER1G,SYK
0	Immune system	Adaptive_NK	PMAIP1	KLRC1,FCER1G,SYK
0	Immune system	Adaptive_NK	FKBP2	KLRC1,FCER1G,SYK
0	Immune system	Adaptive_NK	BANF1	KLRC1,FCER1G,SYK
0	Immune system	Adaptive_NK	PAXX	KLRC1,FCER1G,SYK
0	Immune system	Adaptive_NK	TAOK3	KLRC1,FCER1G,SYK
0	Immune system	Adaptive_NK	CFLAR	KLRC1,FCER1G,SYK
0	Immune system	Adaptive_NK	KRT10	KLRC1,FCER1G,SYK

0	Immune system	Adaptive_NK	SELENOF	KLRC1,FCER1G,SYK
0	Immune system	Adaptive_NK	HIPK1	KLRC1,FCER1G,SYK
0	Immune system	Adaptive_NK	BAZ1A	KLRC1,FCER1G,SYK
0	Immune system	Adaptive_NK	UBE2Q1	KLRC1,FCER1G,SYK
0	Immune system	Adaptive_NK	TRAPPC10	KLRC1,FCER1G,SYK
0	Immune system	Adaptive_NK	MORC3	KLRC1,FCER1G,SYK
0	Immune system	Adaptive_NK	AKAP13	KLRC1,FCER1G,SYK
0	Immune system	Adaptive_NK	PHF20	KLRC1,FCER1G,SYK
0	Immune system	Adaptive_NK	EIF3K	KLRC1,FCER1G,SYK
0	Immune system	Adaptive_NK	WASF2	KLRC1,FCER1G,SYK
0	Immune system	Adaptive_NK	ERBIN	KLRC1,FCER1G,SYK
0	Immune system	Adaptive_NK	BZW1	KLRC1,FCER1G,SYK
0	Immune system	Adaptive_NK	LAT	KLRC1,FCER1G,SYK
0	Immune system	Adaptive_NK	SERPINB6	KLRC1,FCER1G,SYK
0	Immune system	Adaptive_NK	DOCK11	KLRC1,FCER1G,SYK
0	Immune system	Adaptive_NK	C1QBP	KLRC1,FCER1G,SYK
0	Immune system	Adaptive_NK	TROVE2	KLRC1,FCER1G,SYK
0	Immune system	Adaptive_NK	LINC00869	KLRC1,FCER1G,SYK
0	Immune system	Adaptive_NK	SELENOW	KLRC1,FCER1G,SYK
0	Immune system	Adaptive_NK	ARRB2	KLRC1,FCER1G,SYK
0	Immune system	Adaptive_NK	TINF2	KLRC1,FCER1G,SYK
0	Immune system	Adaptive_NK	MIB2	KLRC1,FCER1G,SYK
0	Immune system	Adaptive_NK	TCP1	KLRC1,FCER1G,SYK
0	Immune system	Adaptive_NK	CMPK1	KLRC1,FCER1G,SYK
0	Immune system	Adaptive_NK	RNF125	KLRC1,FCER1G,SYK
0	Immune system	Adaptive_NK	ZBTB7A	KLRC1,FCER1G,SYK
0	Immune system	Adaptive_NK	PTPN4	KLRC1,FCER1G,SYK
0	Immune system	Adaptive_NK	PRNP	KLRC1,FCER1G,SYK
0	Immune system	Adaptive_NK	TADA3	KLRC1,FCER1G,SYK
0	Immune system	Adaptive_NK	FERMT3	KLRC1,FCER1G,SYK
0	Immune system	Adaptive_NK	NCR3	KLRC1,FCER1G,SYK
0	Immune system	Adaptive_NK	TMED5	KLRC1,FCER1G,SYK
0	Immune system	Adaptive_NK	GLG1	KLRC1,FCER1G,SYK
0	Immune system	Adaptive_NK	TMEM9B	KLRC1,FCER1G,SYK
0	Immune system	Adaptive_NK	COX8A	KLRC1,FCER1G,SYK
0	Immune system	Adaptive_NK	RAC1	KLRC1,FCER1G,SYK
0	Immune system	Adaptive_NK	SRSF9	KLRC1,FCER1G,SYK
0	Immune system	Adaptive_NK	TTC16	KLRC1,FCER1G,SYK
0	Immune system	Adaptive_NK	CADM1	KLRC1,FCER1G,SYK
0	Immune system	Adaptive_NK	SGCD	KLRC1,FCER1G,SYK
0	Immune system	Adaptive_NK	DCP1B	KLRC1,FCER1G,SYK
0	Immune system	Adaptive_NK	DRAXIN	KLRC1,FCER1G,SYK
0	Immune system	Adaptive_NK	CAMK2N1	KLRC1,FCER1G,SYK
0	Immune system	Adaptive_NK	LOC283177	KLRC1,FCER1G,SYK
0	Immune system	Adaptive_NK	NCAPH	KLRC1,FCER1G,SYK
0	Immune system	Adaptive_NK	MYO6	KLRC1,FCER1G,SYK
0	Immune system	Adaptive_NK	SBK1	KLRC1,FCER1G,SYK
0	Immune system	Adaptive_NK	CCL5	KLRC1,FCER1G,SYK
0	Immune system	Adaptive_NK	RAB11FIP5	KLRC1,FCER1G,SYK
0	Immune system	Adaptive_NK	JAKMIP1	KLRC1,FCER1G,SYK
0	Immune system	Adaptive_NK	CORO2A	KLRC1,FCER1G,SYK
0	Immune system	Adaptive_NK	B3GAT1	KLRC1,FCER1G,SYK
0	Immune system	Adaptive_NK	SATB2	KLRC1,FCER1G,SYK
0	Immune system	Adaptive_NK	PDGFRB	KLRC1,FCER1G,SYK
0	Immune system	Adaptive_NK	CD6	KLRC1,FCER1G,SYK
0	Immune system	Adaptive_NK	EPB41L4A	KLRC1,FCER1G,SYK
0	Immune system	Adaptive_NK	GDPD5	KLRC1,FCER1G,SYK
0	Immune system	Adaptive_NK	F8	KLRC1,FCER1G,SYK

0	Immune system	Adaptive_NK	LMTK3	KLRC1,FCER1G,SYK
0	Immune system	Adaptive_NK	RCAN2	KLRC1,FCER1G,SYK
0	Immune system	Adaptive_NK	GOLM1	KLRC1,FCER1G,SYK
0	Immune system	Adaptive_NK	ITPRIPL1	KLRC1,FCER1G,SYK
0	Immune system	Adaptive_NK	NUAK1	KLRC1,FCER1G,SYK
0	Immune system	Adaptive_NK	KLRAP1	KLRC1,FCER1G,SYK
0	Immune system	Adaptive_NK	TRG-AS1	KLRC1,FCER1G,SYK
0	Immune system	Adaptive_NK	PPFIA3	KLRC1,FCER1G,SYK
0	Immune system	Adaptive_NK	WNT10B	KLRC1,FCER1G,SYK
0	Immune system	Adaptive_NK	TPRG1	KLRC1,FCER1G,SYK
0	Immune system	Adaptive_NK	CCDC85C	KLRC1,FCER1G,SYK
0	Immune system	Adaptive_NK	LINC00944	KLRC1,FCER1G,SYK
0	Immune system	Adaptive_NK	NSG1	KLRC1,FCER1G,SYK
0	Immune system	Adaptive_NK	CDKN2A	KLRC1,FCER1G,SYK
0	Immune system	Adaptive_NK	DUSP8	KLRC1,FCER1G,SYK
0	Immune system	Adaptive_NK	ACTA2	KLRC1,FCER1G,SYK
0	Immune system	Adaptive_NK	CD2	KLRC1,FCER1G,SYK
0	Immune system	Adaptive_NK	PTMS	KLRC1,FCER1G,SYK
0	Immune system	Adaptive_NK	ABCD2	KLRC1,FCER1G,SYK
0	Immune system	Adaptive_NK	TP53TG1	KLRC1,FCER1G,SYK
0	Immune system	Adaptive_NK	LRRC16B	KLRC1,FCER1G,SYK
0	Immune system	Adaptive_NK	KIAA1671	KLRC1,FCER1G,SYK
0	Immune system	Adaptive_NK	PAK6	KLRC1,FCER1G,SYK
0	Immune system	Adaptive_NK	MXRA7	KLRC1,FCER1G,SYK
0	Immune system	Adaptive_NK	FAM131B	KLRC1,FCER1G,SYK
0	Immune system	Adaptive_NK	ATP8B2	KLRC1,FCER1G,SYK
0	Immune system	Adaptive_NK	FAM53B	KLRC1,FCER1G,SYK
0	Immune system	Adaptive_NK	GLB1L2	KLRC1,FCER1G,SYK
0	Immune system	Adaptive_NK	CDC14B	KLRC1,FCER1G,SYK
0	Immune system	Adaptive_NK	PCNXL2	KLRC1,FCER1G,SYK
0	Immune system	Adaptive_NK	HOXC5	KLRC1,FCER1G,SYK
0	Immune system	Adaptive_NK	MCOLN2	KLRC1,FCER1G,SYK
0	Immune system	Adaptive_NK	MVB12B	KLRC1,FCER1G,SYK
0	Immune system	Adaptive_NK	ZNF365	KLRC1,FCER1G,SYK
0	Immune system	Adaptive_NK	SOX13	KLRC1,FCER1G,SYK
0	Immune system	Adaptive_NK	GCNT4	KLRC1,FCER1G,SYK
0	Immune system	Adaptive_NK	KLRC3	KLRC1,FCER1G,SYK
0	Immune system	Adaptive_NK	LAG3	KLRC1,FCER1G,SYK
0	Immune system	Adaptive_NK	TMEM255A	KLRC1,FCER1G,SYK
0	Immune system	Adaptive_NK	GOLGA7B	KLRC1,FCER1G,SYK
0	Immune system	Adaptive_NK	NINL	KLRC1,FCER1G,SYK
0	Immune system	Adaptive_NK	DAPK2	KLRC1,FCER1G,SYK
0	Immune system	Adaptive_NK	ARHGEF28	KLRC1,FCER1G,SYK
0	Immune system	Adaptive_NK	GNAO1	KLRC1,FCER1G,SYK
0	Immune system	Adaptive_NK	PBX4	KLRC1,FCER1G,SYK
0	Immune system	Adaptive_NK	CRIP1	KLRC1,FCER1G,SYK
0	Immune system	Adaptive_NK	EPB41L4A-AS2	KLRC1,FCER1G,SYK
0	Immune system	Adaptive_NK	LINC00943	KLRC1,FCER1G,SYK
0	Immune system	Adaptive_NK	PATL2	KLRC1,FCER1G,SYK
0	Immune system	Adaptive_NK	FKBP1B	KLRC1,FCER1G,SYK
0	Immune system	Adaptive_NK	MLF1	KLRC1,FCER1G,SYK
0	Immune system	Adaptive_NK	EPN2	KLRC1,FCER1G,SYK
0	Immune system	Adaptive_NK	KIF5A	KLRC1,FCER1G,SYK
0	Immune system	Adaptive_NK	KCNA3	KLRC1,FCER1G,SYK
0	Immune system	Adaptive_NK	LRFN2	KLRC1,FCER1G,SYK
0	Immune system	Adaptive_NK	ISL2	KLRC1,FCER1G,SYK
0	Immune system	Adaptive_NK	LIME1	KLRC1,FCER1G,SYK
0	Immune system	Adaptive_NK	GPR153	KLRC1,FCER1G,SYK

0	Immune system	Adaptive_NK	KLHL4	KLRC1,FCER1G,SYK
0	Immune system	Adaptive_NK	KIAA1324	KLRC1,FCER1G,SYK
0	Immune system	Adaptive_NK	VSTM2B	KLRC1,FCER1G,SYK
0	Immune system	Adaptive_NK	CDYL2	KLRC1,FCER1G,SYK
0	Immune system	Adaptive_NK	SLC35G2	KLRC1,FCER1G,SYK
0	Immune system	Adaptive_NK	ATL1	KLRC1,FCER1G,SYK
0	Immune system	Adaptive_NK	FRMPD3	KLRC1,FCER1G,SYK
0	Immune system	Adaptive_NK	CXXC4	KLRC1,FCER1G,SYK
0	Immune system	Adaptive_NK	CLTCL1	KLRC1,FCER1G,SYK
0	Immune system	Adaptive_NK	FAM167A	KLRC1,FCER1G,SYK
0	Immune system	Adaptive_NK	PPP2R2B	KLRC1,FCER1G,SYK
0	Immune system	Adaptive_NK	LRFN3	KLRC1,FCER1G,SYK
0	Immune system	Adaptive_NK	HOXC4	KLRC1,FCER1G,SYK
0	Immune system	Adaptive_NK	TCERG1L	KLRC1,FCER1G,SYK
0	Immune system	Adaptive_NK	C1orf177	KLRC1,FCER1G,SYK
0	Immune system	Adaptive_NK	TSHZ3	KLRC1,FCER1G,SYK
0	Immune system	Adaptive_NK	FOXD1	KLRC1,FCER1G,SYK
0	Immune system	Adaptive_NK	MYO3B	KLRC1,FCER1G,SYK
0	Immune system	Adaptive_NK	TMEM244	KLRC1,FCER1G,SYK
0	Immune system	Adaptive_NK	IER5L	KLRC1,FCER1G,SYK
0	Immune system	Adaptive_NK	RASGEF1A	KLRC1,FCER1G,SYK
0	Immune system	Adaptive_NK	MID2	KLRC1,FCER1G,SYK
0	Immune system	Adaptive_NK	CD3D	KLRC1,FCER1G,SYK
0	Immune system	Adaptive_NK	SELM	KLRC1,FCER1G,SYK
0	Immune system	Adaptive_NK	TLR3	KLRC1,FCER1G,SYK
0	Immune system	Adaptive_NK	RAB6B	KLRC1,FCER1G,SYK
0	Immune system	Adaptive_NK	LRRC75A	KLRC1,FCER1G,SYK
0	Immune system	Adaptive_NK	MIR4435-2HG	KLRC1,FCER1G,SYK
0	Immune system	Adaptive_NK	KLRC4	KLRC1,FCER1G,SYK
0	Immune system	Adaptive_NK	LTBP4	KLRC1,FCER1G,SYK
0	Immune system	Adaptive_NK	PERP	KLRC1,FCER1G,SYK
0	Immune system	Adaptive_NK	SCN8A	KLRC1,FCER1G,SYK
0	Immune system	Adaptive_NK	DUSP19	KLRC1,FCER1G,SYK
0	Immune system	Adaptive_NK	EDNRB-AS1	KLRC1,FCER1G,SYK
0	Immune system	Adaptive_NK	CRYBB3	KLRC1,FCER1G,SYK
0	Immune system	Adaptive_NK	SCD5	KLRC1,FCER1G,SYK
0	Immune system	Adaptive_NK	LOC102724094	KLRC1,FCER1G,SYK
0	Immune system	Adaptive_NK	ELOVL4	KLRC1,FCER1G,SYK
0	Immune system	Adaptive_NK	SPATA6L	KLRC1,FCER1G,SYK
0	Immune system	Adaptive_NK	TF	KLRC1,FCER1G,SYK
0	Immune system	Adaptive_NK	EFNA5	KLRC1,FCER1G,SYK
0	Immune system	Adaptive_NK	SLC45A1	KLRC1,FCER1G,SYK
0	Immune system	Adaptive_NK	CDKN2B-AS1	KLRC1,FCER1G,SYK
0	Immune system	Adaptive_NK	OTOF	KLRC1,FCER1G,SYK
0	Immune system	Adaptive_NK	C1orf61	KLRC1,FCER1G,SYK
0	Immune system	Adaptive_NK	CHRNE	KLRC1,FCER1G,SYK
0	Immune system	Adaptive_NK	TKTL1	KLRC1,FCER1G,SYK
0	Immune system	Adaptive_NK	TSPAN2	KLRC1,FCER1G,SYK
0	Immune system	Adaptive_NK	LOC101928988	KLRC1,FCER1G,SYK
0	Immune system	Adaptive_NK	PARD6G	KLRC1,FCER1G,SYK
0	Immune system	Adaptive_NK	STXBP6	KLRC1,FCER1G,SYK
0	Immune system	Adaptive_NK	TRPC3	KLRC1,FCER1G,SYK
0	Immune system	Adaptive_NK	JAKMIP2	KLRC1,FCER1G,SYK
0	Immune system	Adaptive_NK	HEY2	KLRC1,FCER1G,SYK
0	Immune system	Adaptive_NK	TRIM46	KLRC1,FCER1G,SYK
0	Immune system	Adaptive_NK	SNPH	KLRC1,FCER1G,SYK
0	Immune system	Adaptive_NK	HRASLS5	KLRC1,FCER1G,SYK
0	Immune system	Adaptive_NK	SLC14A2	KLRC1,FCER1G,SYK

0	Immune system	Adaptive_NK	KRTAP5-AS1	KLRC1,FCER1G,SYK
0	Immune system	Adaptive_NK	WWTR1	KLRC1,FCER1G,SYK
0	Immune system	Adaptive_NK	SPTBN5	KLRC1,FCER1G,SYK
0	Immune system	Adaptive_NK	KIR2DL2	KLRC1,FCER1G,SYK
0	Immune system	Adaptive_NK	SLC14A1	KLRC1,FCER1G,SYK
0	Immune system	Adaptive_NK	C16orf45	KLRC1,FCER1G,SYK
0	Immune system	Adaptive_NK	TJP3	KLRC1,FCER1G,SYK
0	Immune system	Adaptive_NK	ATP1A3	KLRC1,FCER1G,SYK
0	Immune system	Adaptive_NK	ST8SIA1	KLRC1,FCER1G,SYK
0	Immune system	Adaptive_NK	AGAP1	KLRC1,FCER1G,SYK
0	Immune system	Adaptive_NK	KCCAT198	KLRC1,FCER1G,SYK
0	Immune system	Adaptive_NK	PTPRM	KLRC1,FCER1G,SYK
0	Immune system	Adaptive_NK	NFIA	KLRC1,FCER1G,SYK
0	Immune system	Adaptive_NK	DGKH	KLRC1,FCER1G,SYK
0	Immune system	Adaptive_NK	GREB1	KLRC1,FCER1G,SYK
0	Immune system	Adaptive_NK	LINC00565	KLRC1,FCER1G,SYK
0	Immune system	Adaptive_NK	NUGGC	KLRC1,FCER1G,SYK
0	Immune system	Adaptive_NK	TUBB4A	KLRC1,FCER1G,SYK
0	Immune system	Adaptive_NK	IL5RA	KLRC1,FCER1G,SYK
0	Immune system	Adaptive_NK	SGCB	KLRC1,FCER1G,SYK
0	Immune system	Adaptive_NK	NPR3	KLRC1,FCER1G,SYK
0	Immune system	Adaptive_NK	DENND2C	KLRC1,FCER1G,SYK
0	Immune system	Adaptive_NK	CD52	KLRC1,FCER1G,SYK
0	Immune system	Adaptive_NK	CDC14C	KLRC1,FCER1G,SYK
0	Immune system	Adaptive_NK	MUC3A	KLRC1,FCER1G,SYK
0	Immune system	Adaptive_NK	ERRFI1	KLRC1,FCER1G,SYK
0	Immune system	Adaptive_NK	LILRB1	KLRC1,FCER1G,SYK
0	Immune system	Adaptive_NK	IMPG1	KLRC1,FCER1G,SYK
0	Immune system	Adaptive_NK	GSC	KLRC1,FCER1G,SYK
0	Immune system	Adaptive_NK	SCUBE3	KLRC1,FCER1G,SYK
0	Immune system	Adaptive_NK	WNT1	KLRC1,FCER1G,SYK
0	Immune system	Adaptive_NK	EPHX2	KLRC1,FCER1G,SYK
0	Immune system	Adaptive_NK	MAMLD1	KLRC1,FCER1G,SYK
0	Immune system	Adaptive_NK	NACAD	KLRC1,FCER1G,SYK
0	Immune system	Adaptive_NK	LAMB1	KLRC1,FCER1G,SYK
0	Immune system	Adaptive_NK	CHSY3	KLRC1,FCER1G,SYK
0	Immune system	Adaptive_NK	SHB	KLRC1,FCER1G,SYK
0	Immune system	Adaptive_NK	ADAMTSL5	KLRC1,FCER1G,SYK
0	Immune system	Adaptive_NK	LOC101929719	KLRC1,FCER1G,SYK
0	Immune system	Adaptive_NK	TRPC1	KLRC1,FCER1G,SYK
0	Immune system	Adaptive_NK	IL32	KLRC1,FCER1G,SYK
0	Immune system	Adaptive_NK	ZFP36	KLRC1,FCER1G,SYK
0	Immune system	Adaptive_NK	WDR74	KLRC1,FCER1G,SYK
0	Immune system	Adaptive_NK	CD160	KLRC1,FCER1G,SYK
0	Immune system	Adaptive_NK	XCL2	KLRC1,FCER1G,SYK
0	Immune system	Adaptive_NK	ID2	KLRC1,FCER1G,SYK
0	Immune system	Adaptive_NK	IER2	KLRC1,FCER1G,SYK
0	Immune system	Adaptive_NK	CD7	KLRC1,FCER1G,SYK
0	Immune system	Adaptive_NK	SNORD3A	KLRC1,FCER1G,SYK
0	Immune system	Adaptive_NK	CCL3	KLRC1,FCER1G,SYK
0	Immune system	Adaptive_NK	TMEM107	KLRC1,FCER1G,SYK
0	Immune system	Adaptive_NK	FOS	KLRC1,FCER1G,SYK
0	Immune system	Adaptive_NK	XCL1	KLRC1,FCER1G,SYK
0	Immune system	Adaptive_NK	SNORD3D	KLRC1,FCER1G,SYK
0	Immune system	Adaptive_NK	MAP3K8	KLRC1,FCER1G,SYK
0	Immune system	Adaptive_NK	JUN	KLRC1,FCER1G,SYK
0	Immune system	Adaptive_NK	PPP1R15A	KLRC1,FCER1G,SYK
0	Immune system	Adaptive_NK	VIM	KLRC1,FCER1G,SYK

0	Immune system	Adaptive_NK	RNU12	KLRC1,FCER1G,SYK
0	Immune system	Adaptive_NK	SNORD3B-1	KLRC1,FCER1G,SYK
0	Immune system	Adaptive_NK	IL2RB	KLRC1,FCER1G,SYK
0	Immune system	Adaptive_NK	TMIGD2	KLRC1,FCER1G,SYK
0	Immune system	Adaptive_NK	RP11-386I14.4	KLRC1,FCER1G,SYK
0	Immune system	Adaptive_NK	GZMK	KLRC1,FCER1G,SYK
0	Immune system	Adaptive_NK	LTB	KLRC1,FCER1G,SYK
