# Supplementary figures and images for: PRDM1 Is Associated with Chemoradiotherapy-Associated Enrichment of Adaptive NK Cells in Cervical Cancer
Source: Comput Struct Biotechnol J. 2026 May 7;35(1):0092. doi: 10.34133/csbj.0092 (PMC13150072; doi:10.34133/csbj.0092)

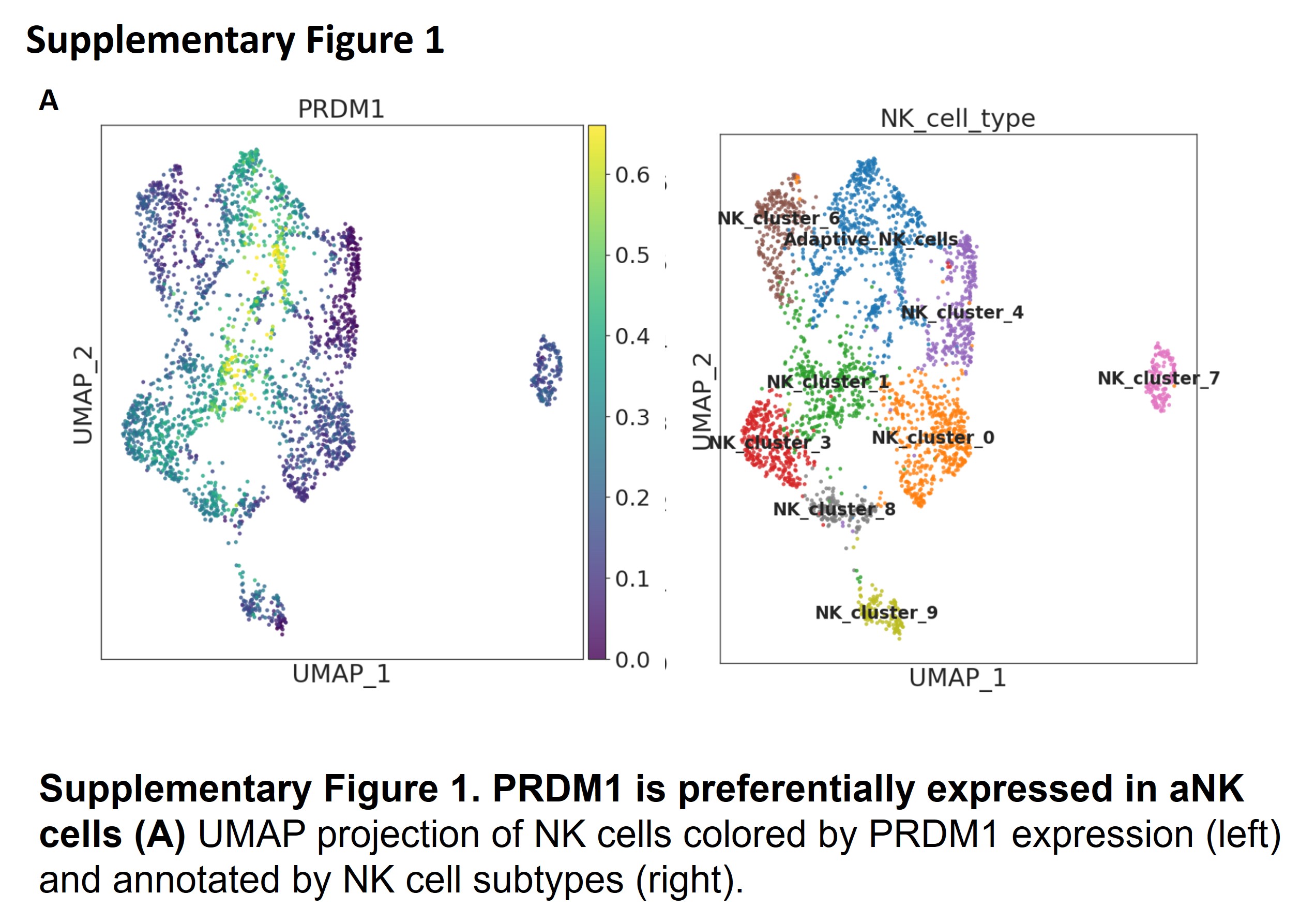

Supplement: Supplementary 1 — Figs. S1 to S3 Table S1 [file csbj.0092.f1.zip › Supplementary Figure 1.jpg]

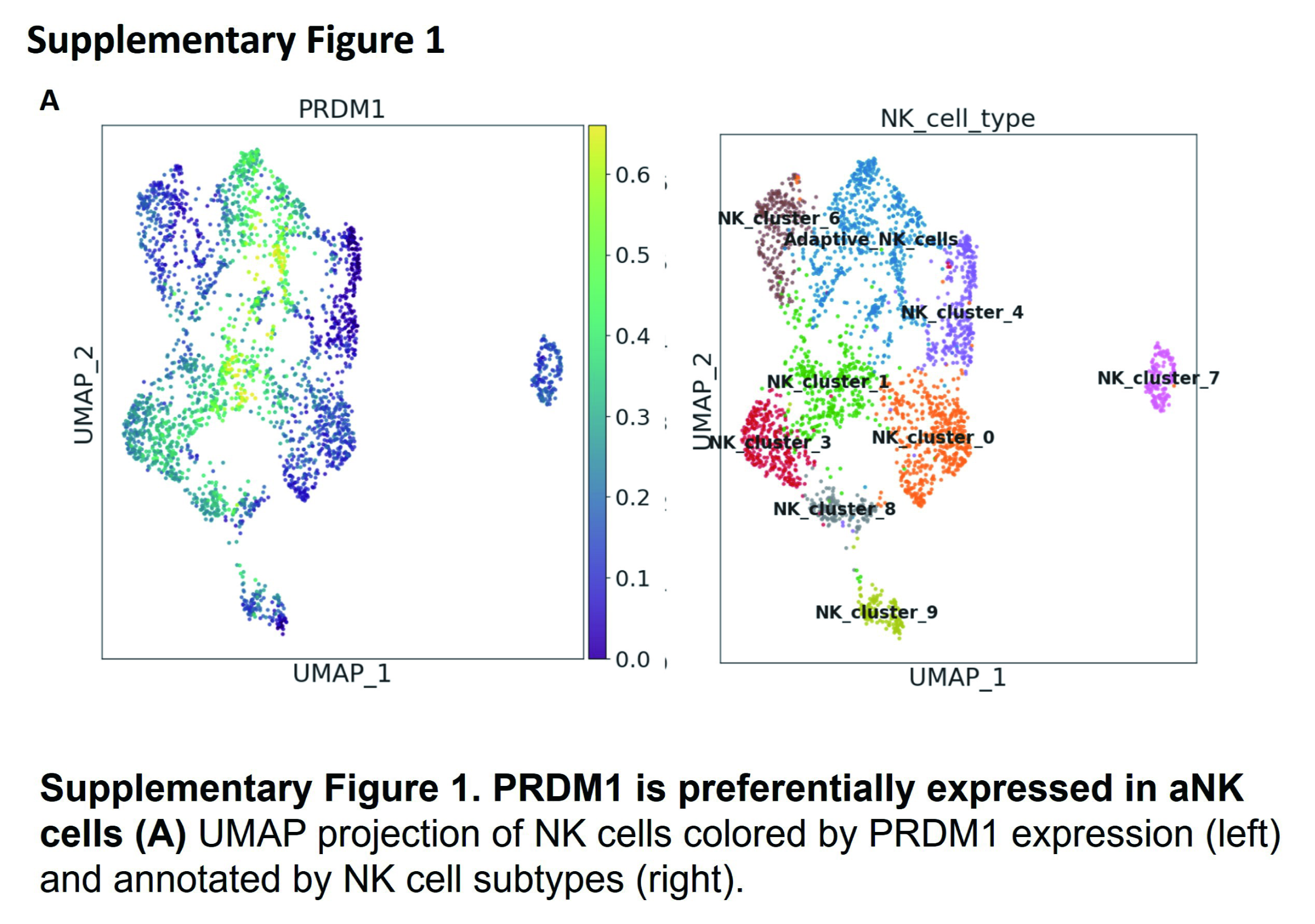

Supplement: Supplementary 1 — Figs. S1 to S3 Table S1 [file csbj.0092.f1.zip › Supplementary Figure 1.tif]

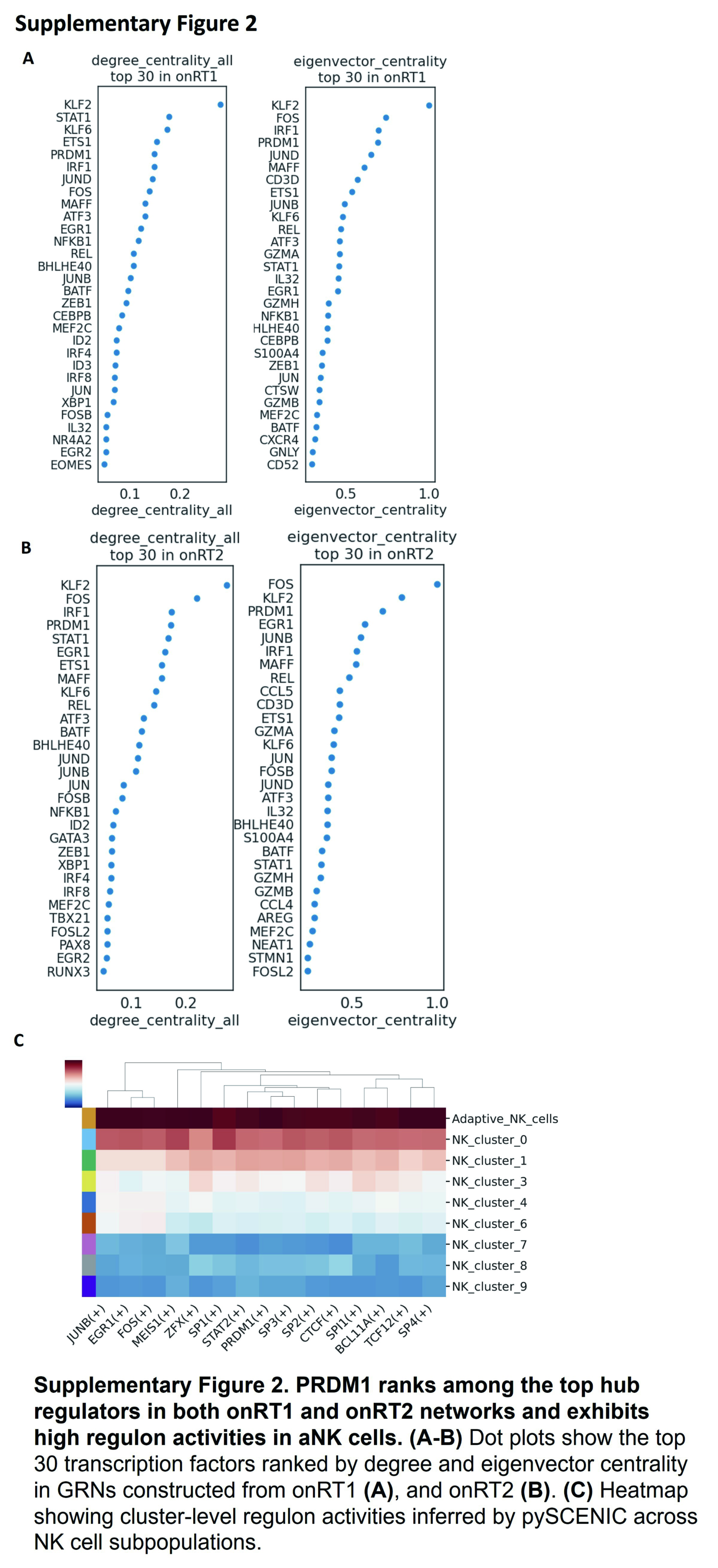

Supplement: Supplementary 1 — Figs. S1 to S3 Table S1 [file csbj.0092.f1.zip › Supplementary Figure 2.tif]

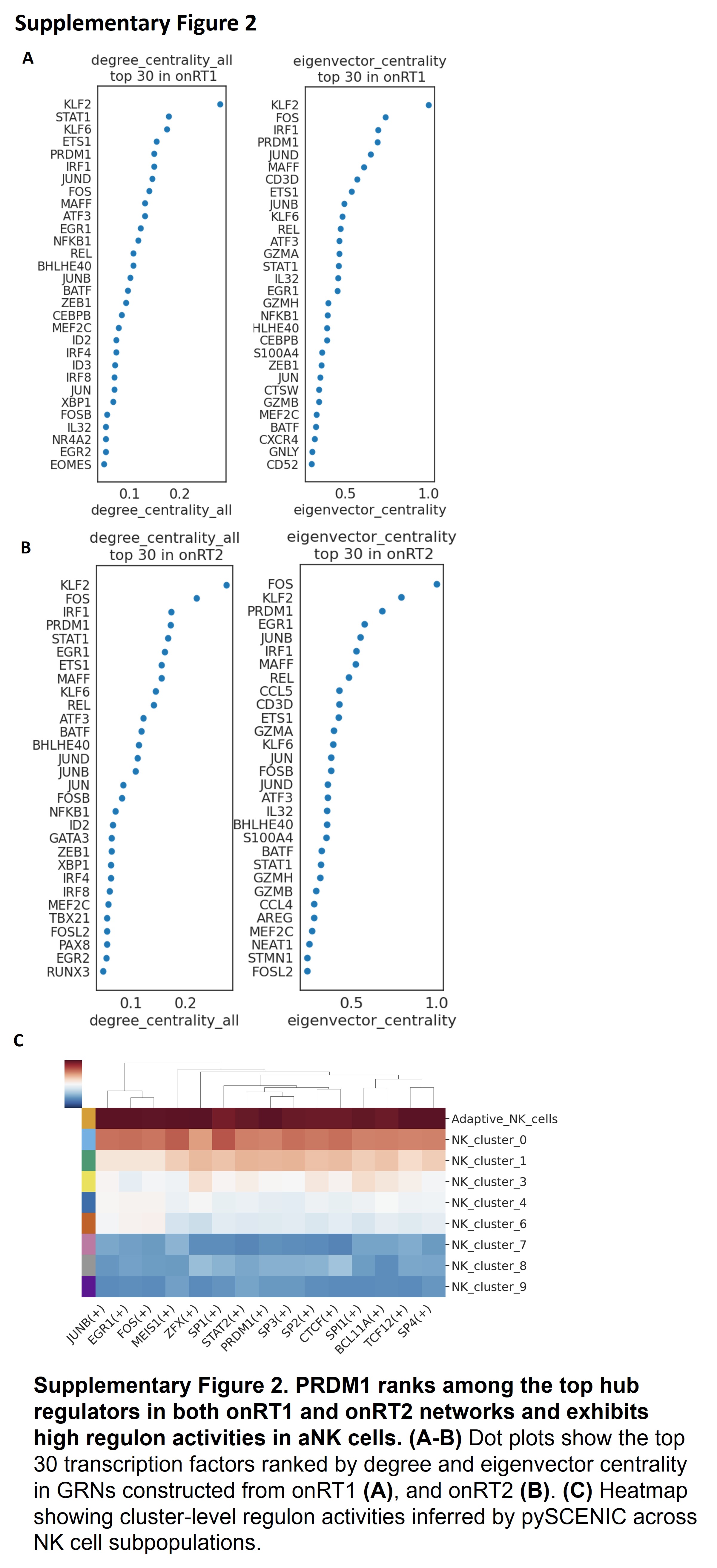

Supplement: Supplementary 1 — Figs. S1 to S3 Table S1 [file csbj.0092.f1.zip › Supplementary Figure 2_revised.jpg]

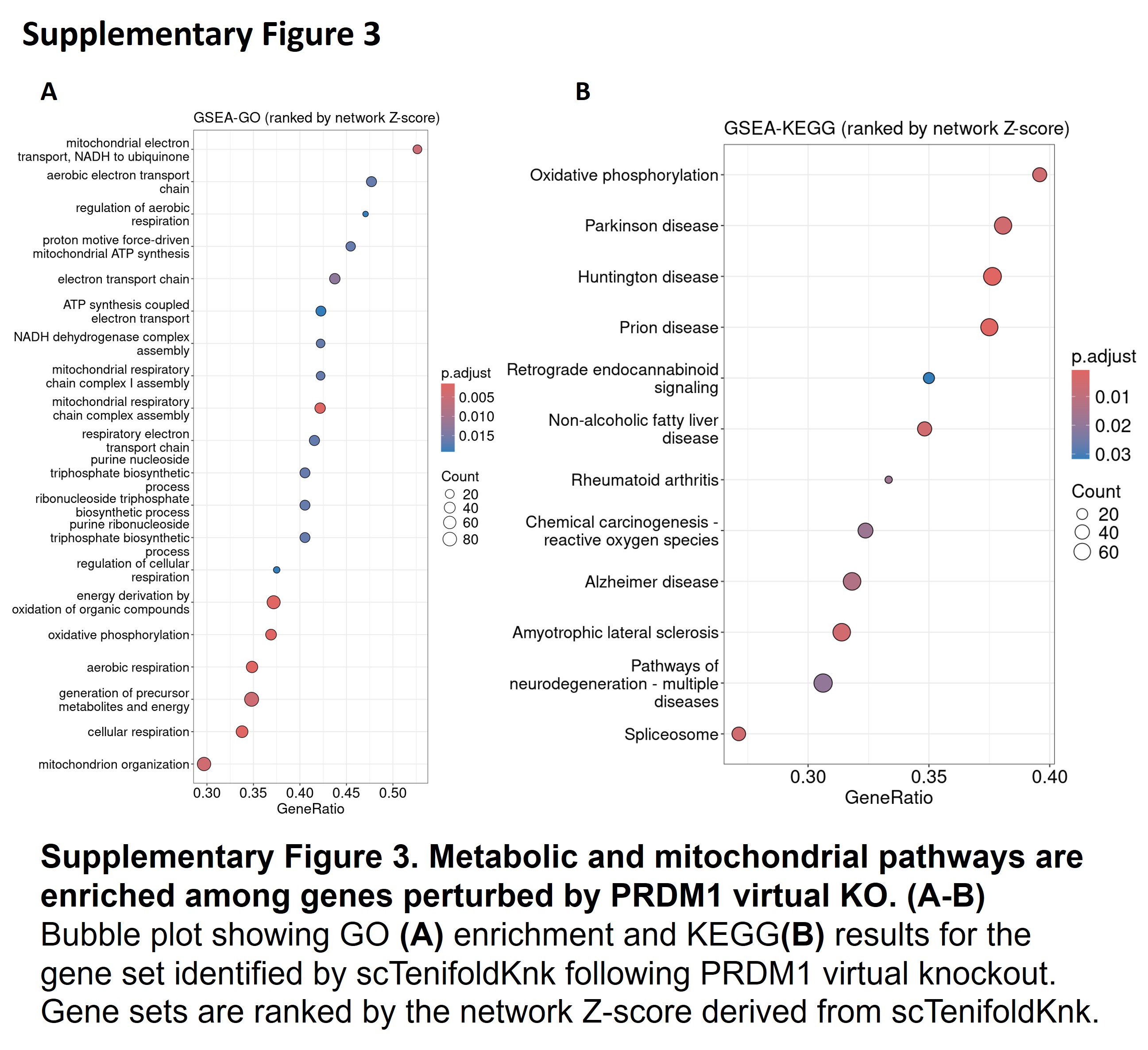

Supplement: Supplementary 1 — Figs. S1 to S3 Table S1 [file csbj.0092.f1.zip › Supplementary Figure 3.jpg]

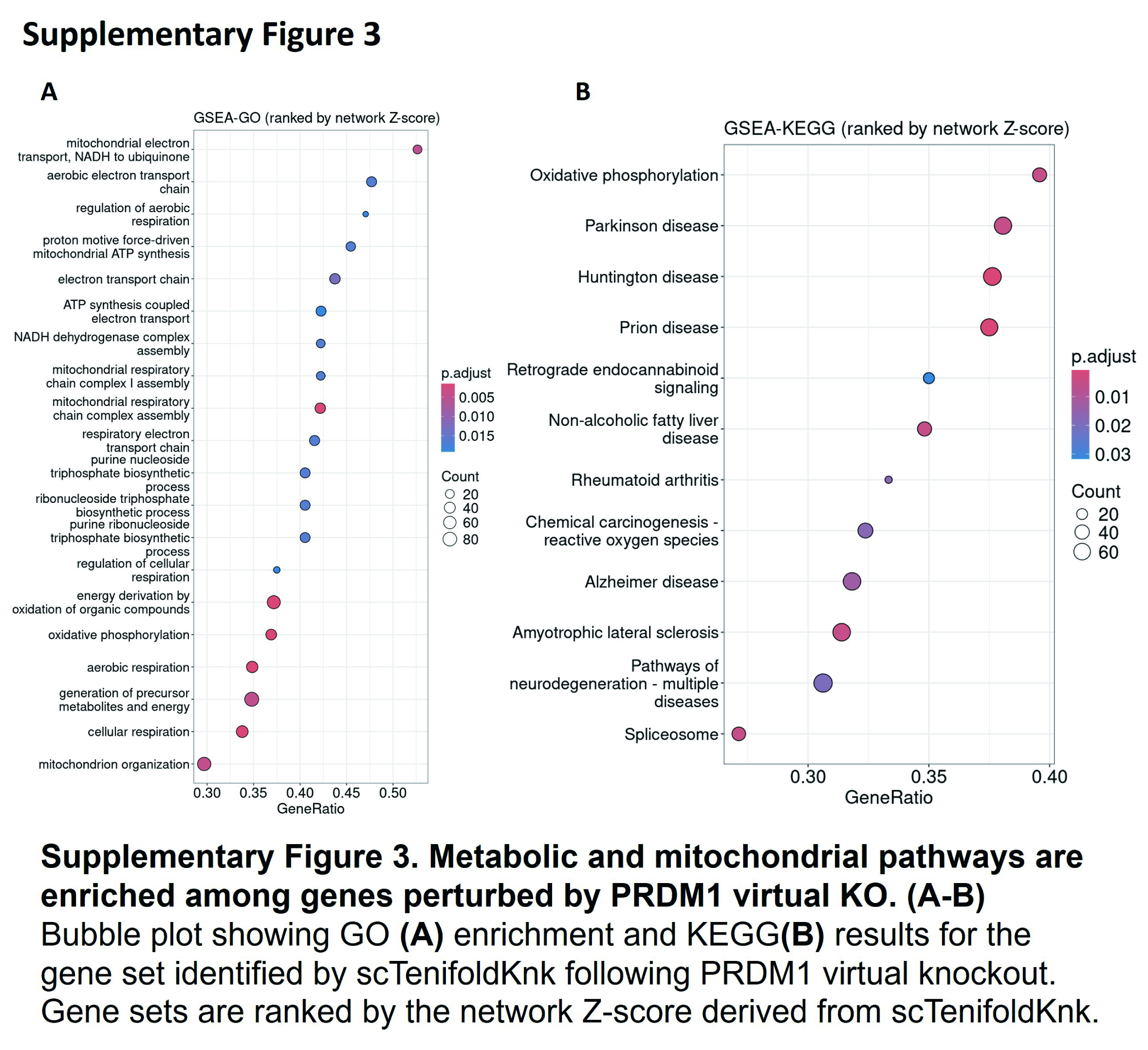

Supplement: Supplementary 1 — Figs. S1 to S3 Table S1 [file csbj.0092.f1.zip › Supplementary Figure 3.tif]
